# Isoform‐specific effects of transcription factor TCFL5 on the pluripotency‐related genes SOX2 and KLF4 in colorectal cancer development

**DOI:** 10.1002/1878-0261.13085

**Published:** 2021-10-08

**Authors:** Javier Galán‐Martínez, Konstantinos Stamatakis, Inés Sánchez‐Gómez, Silvia Vázquez‐Cuesta, Núria Gironés, Manuel Fresno

**Affiliations:** ^1^ Centro de Biología Molecular Severo Ochoa Universidad Autónoma de Madrid Spain; ^2^ Consejo Superior de Investigaciones Científicas Universidad Autónoma de Madrid Spain; ^3^ Instituto Sanitario de Investigación Princesa Madrid Spain

**Keywords:** colorectal cancer, isoforms, KLF4, SOX2, TCFL5

## Abstract

Colorectal cancer (CRC) is a very common life‐threatening malignancy. Transcription factor‐like 5 (TCFL5) has been suggested to be involved in CRC. Here, we describe the expression of four alternative transcripts of TCFL5 and their relevance in CRC. Complete deletion of all isoforms drastically decreased pro‐tumoural properties such as spheroids formation and *in vivo* tumour growth, although increased migration in CRC cell lines. Overexpression of the two main isoforms, TCFL5_E8 and CHA, had opposite effects: TCFL5_E8 reduced proliferation and spheroids formation, while CHA increased them. TCFL5_E8 reduced *in vivo* tumour formation, while CHA had no effect. In addition, TCFL5_E8 and CHA have different roles in the regulation of the pluripotency‐related genes *SOX2* and *KLF4*. Both isoforms bind directly to their promoters; however, TCFL5_E8 induced *SOX2* and reduced *KLF4* mRNA levels, whereas CHA did the opposite. Together, our results show that *TCFL5* plays an important role in the development of CRC, being however isoform‐specific. This work also points to the need to analyse separately TCFL5 isoforms in cancer, due to their different and opposite functions.

## Graphical abstract

In this study, the authors identify four isoforms of TCFL5 in colorectal cancer, each with strikingly different properties. Complete deletion of the *TCFL5* locus reduced tumour cell proliferation and spheroid formation, but increased migration. However, whereas overexpression of the TCFL5_E8 isoform reduced proliferation and spheroid formation, overexpression of the CHA isoform increased them. In addition, CHA and TCFL5_E8 bound to the *SOX2* and *KLF4* promoters directly, controlling their transcription.

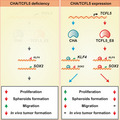

**Abbreviations**
APC: adenomatous polyposis coli
bFGF: basic fibroblast growth factor
bHLH: basic Helix Loop Helix
CD2: T‐cell surface antigen CD2
CLGN: calmegina
CRC: colorectal cancer
CRISPR: clustered regularly interspaced short palindromic repeats
CSC: cancer stem cell
EGF: epidermal growth factor
EMT: epithelial–mesenchymal transition
HSP90: heat shock protein 90
KLF4: Kruppel‐like factor 4
KO: knockout
MCTS: multicellular tumour spheroids
MEM: minimum essential medium eagle
PFA: paraformaldehyde
PIK3CA: phosphatidylinositol‐4,5‐bisphosphate 3‐kinase catalytic subunit alpha
PTEN: phosphatase and tensin homolog
qPCR: quantitative PCR
RT: room temperature
shRNA: small hairpain RNA
siRNA: small interfering RNA
SOX2: SRY (Sex‐Determining Region Y)‐Box 2
TBST: Tris‐buffered saline–Tween
TCFL5: transcription factor‐like 5
TFs: transcription factors
TP53: tumour protein p53
USF‐1: upstream transcription factor 1
WT: wild‐type

## 1 Introduction

Cancer is the main cause of death in developed countries killing around 10 million people a year [1]. Colorectal cancer (CRC) is the second most common type of cancer in both men and women [2]. Several treatments such as surgery, chemotherapy and radiotherapy are used in CRC. However, in many cases, treatments are not entirely effective, urging the need for new approaches to fight against CRC [3]. Transcription factors (TFs) are key regulators of gene expression. They have an important role in several cellular processes, and their dysregulation is associated with many pathological processes such as cancer [4]. Changes in the transcriptional network have been associated with TFs expression deregulation [5, 6, 7, 8] proving that these proteins are essential for cancer development. For this reason, TFs are the target of several antitumour strategies [9, 10].

Transcription Factor‐like 5 (TCFL5) is a scarcely studied TF of the basic Helix Loop Helix (bHLH) family. TCFL5 was first described in testis, specifically in spermatocytes [11]. In spermatogenesis, TCFL5 acts as a TF controlling the expression of *CLGN*, although it has also been described in the manchette of spermatid co‐localizing with tubulin [12, 13]. In addition, TCFL5 is a target of the NOTCH pathway in T cells [14]. *TCFL5* mRNA induction during activation or development of myeloid and lymphoid cells has been reported [14, 15, 16]. CHA, a shorter isoform of TCFL5, encoded by the same gene, was described as a partner of USF‐1 in leukaemic T lymphocytes playing a role in T‐cell activation and inhibiting CD2 expression [17]. Both isoforms share the bHLH domain and only differ in the first exon. However, its exact function is still mostly unknown.

In cancer, *TCFL5* is upregulated in leukaemia and seminomas [18, 19, 20]. In CRC, TCFL5 expression has been found to be higher in carcinomas than in adenomas, likely due to amplification in chromosome 20q [21]. Moreover, in HT29 colon carcinoma cell line induction of TCFL5 during multicellular tumour spheroids (*MCTS*) formation, an *in vitro* cellular aggregation model which mimics tumour formation [22, 23] has been described [24]. However, none of the above studies discriminated between TCFL5 and CHA isoforms.

Here, we report for the first time the expression of four TCFL5 isoforms in CRC cell lines. The complete deletion of *TCFL5* locus produced a remarkable decrease in the tumoural properties of CRC cell lines. Consequently, the function of the most relevant isoforms, TCFL5_E8 and CHA, was studied more in detail in a CRC cell line. These isoforms exhibited different and mostly opposite effects on several tumoural properties such as proliferation, migration, spheroids formation and *in vivo* tumour formation. Finally, TCFL5_E8 and CHA were found to control the expression of master pluripotency markers SOX2 and KLF4 by directly binding to their promoters.

## 2 Material & methods

### 2.1 Cell lines

Human HCT116 cell line was obtained from Centro de Investigaciones Biológicas (Madrid, Spain). SW480 and SW620 cell lines were obtained from Instituto de Investigaciones Biomédicas (Madrid, Spain). HT29lucD6 cell line was obtained from Xenogen Corporation. HEK‐293T cell line was obtained from Centro de Biología Molecular ‘Severo Ochoa’ (Madrid, Spain). Cells were obtained and grown as described [25]. HCT116 TCFL5^−/−^cell line was generated by Self‐cloning CRISPR‐Cas9 technique following the established protocol [26]. Two guides flanking exon E3 were used to remove this exon (Table S1). Those guides were amplified by PCR according to the protocol (Table S1). HCT116 cells (3 x 10^5^ cells) were transfected with 10 µL of both amplified guides, 0.5 µg psqPal plasmid and 0.5 µg pCas9 plasmid using Metafectene Pro (Biontex, München, Germany) according to manufacturer’s instructions. Transfected HCT116 was selected after 48 h with 10 µg·mL^−1^ blasticidin (Invitrogen, Carlsbad, CA, USA) and 200 µg·mL^−1^ hygromycin B (Invitrogen) for 48 h. Resulting cells were sorted using FACSVantage SE (BD Bioscience, Franklin Lakes, NJ, USA). Clones were sequenced and validated by PCR (Table S1). CHA and TCFL5 overexpressing HCT116 and HT29lucD6 cell lines were generated by lentiviral particle transduction carrying construct gene or empty vector as previously described [27]. Human CHA and TCFL5 isoforms were subcloned in the pLenti‐CMV/TO‐Hygro vector using specific oligonucleotides (Table S1) from pCMV6‐XL5‐TCFL5 (OriGene, Rockville, MD, USA). Cells were selected using 200 µg·mL^−1^ hygromycin B (Invitrogen). Silenced cell lines were obtained transfecting shRNA and siRNA for TCFL5 (Table S1) using Metafectene Pro (Biontex) according to the manufacturer’s instructions.

### 2.2 Multicellular tumour spheroids (spheroid) formation

Cells were seeded in low‐attachment 96‐well or p100 plates at a density of 4 × 10^4^ cells/well in MEM supplemented with 0.4% FBS, 1 ng·mL^−1^ basic fibroblast growth factor (bFGF) (Sigma, San Luis, MO, USA), 10 ng·mL^−1^ epidermal growth factor (EGF) (Sigma), 5 µg·mL^−1^ insulin (Sigma) and 1x B27 (Invitrogen). Spheroids were cultured for 7 days and used for RNA and protein analysis, or size and number analysis. For size and number analysis, spheroid images were taken using Leica DM IL microscopy (Leica Microsystems, Wetzlar, Germany). Images were quantified by ImageJ software (National Institutes of Health, NIH).

### 2.3 Proliferation and colony assays

Cell number proliferation determination was performed as described [25]. For Alamar Blue assay, 5 × 10^3^ cells·well^−1^ were seeded in 96‐well plates. After 48 h, proliferation was quantified using alamarBlue (Thermofisher, Waltham, MA, USA) according to the manufacturer’s instructions. Fluorescence was measured in a FLUOstar OPTIMA plate reader (BMG LABTECH, Ortenberg, Germany). For colony assay, 500 cells/well were seeded in 6‐well plates. After 10 days, cells were fixed in 4% paraformaldehyde (PFA) for 20 min at room temperature (RT). Cells were stained using Crystal Violet (50% H_2_Od‐methanol and 0.5% crystal violet) for 30 min at RT. Colonies were counted manually.

### 2.4 Wound healing assay

Cells were seeded in 6‐well plates at 80% of confluence in low FBS medium (0.4% FBS – complete MEM). A 0.4‐mm wide wound was performed after cells were attached to the plate using a 10 µL tip. Wound images were taken during three days using Leica DM IL microscopy (Leica Microsystems). Images were quantified with ImageJ software (National Institutes of Health, NIH). Wound healing area was normalized to time 0.

### 2.5 Tumour xenografts assay

Swiss Nude (Crl : NU(Ico)‐Foxn1^nu^) mice (8 weeks of age) purchased from Charles River Laboratory were maintained in an animal‐biosafety level 2 room under a specific pathogen‐free environment. HCT116‐ and HT29lucD6‐derived cell lines were injected subcutaneously (1.0 × 10^6^ cell/mouse) in females using 5 mice per group. Tumour growth was studied every week for 5–8 weeks. Tumours were measured using a handheld caliper.

All animal procedures were performed in strict accordance with the European Commission legislation for the protection of animals (2010/63/EU). The protocol for the treatment of the animals was approved by the Comité de Ética de la Dirección General del Medio Ambiente de la Comunidad de Madrid, Spain (permits PROEX 240/19) and was supervised by the Ethics Committee of CBMSO (Madrid, Spain).

### 2.6 Immunoblot and RNA analysis

Immunoblot was performed as described [25]. For specific protein detection, membranes were incubated with anti‐TCFL5 (Sigma), anti‐SOX2 (Cell Signalling, Danvers, MA, USA), anti‐KLF4 (Cell Signalling), anti‐E‐cadherin (Cell Signaling) and anti‐HSP90 (Sigma) in 5% BSA‐TBST overnight at 4 °C. Complete membranes were shown in Fig. S6.

RNA was isolated and reverse transcribed as described [25]. cDNA was used both for PCR using GoTaq Flexi DNA Polymerase (Promega, Madison, WI, USA) or quantitative PCR (qPCR) using GoTaq 1PCR Master Mix (Promega) with specific primers (Table S1), according to the manufacturer’s instructions. For qPCR, values were normalized as described [25].

### 2.7 Chromatin immunoprecipitation assay

Chromatin immunoprecipitation (ChIP) was performed as described [28]. 10^7^ HCT116 cells were transfected with pCDNA3‐TCFL5‐Flag, pCDNA3‐CHA‐Flag and pCDNA3‐EV‐Flag plasmid using Metafectene Pro (Biontex) according to the manufacturer’s instructions, fixed, cross‐linked and lysed. The cross‐linked cell chromatin was sheared by sonication using a Bioruptor Next Gen (Diagentde). Sheared chromatin was incubated with anti‐Flag M2 magnetic beads (Sigma) and immunoprecipitated using QuadroMACS Separator (Miltenyl Biotec). DNA was isolated and analysed by qPCR using specific oligonucleotides (Table S1).

### 2.8 Luciferase reporter activity assay

Cells were grown in 24‐well plates and transfected with pCDNA3‐CHA‐Flag, pCDNA3‐TCFL5‐Flag or pEP4 E02S CK2M EN2L, expression vector carrying the Oct4 and Sox2; Klf4 and Myc; Nanog and Lin28 genes (a gift from James Thomson, Addgene Plasmid#20924) plasmids in combination with pGL4‐SOX2‐CORE, pGL4‐SOX2‐SRR1, pGL4‐KLF4‐RE, pGL4‐4x‐E‐box reporter plasmids and SV40‐Renilla control plasmid using Metafectene Pro (Biontex) according to manufacturer’s instructions. After 48 h, cells were lysed and luciferase activity was measured in a 96‐well Nunclon plate in a FLUOstar OPTIMA plate reader (BMG LABTECH) using Dual‐Luciferase Reporter Assay Kit (Promega) according to manufacturer’s instructions. Relative luciferase activity was obtained using a ratio luciferase/renilla and samples/control.

### 2.9 Statistics

Statistical analysis was performed using graphpad Prism v6.0 software (GraphPad Software, LLC). Results were expressed as means ± SEM (Standard Error of the Mean). Statistical method used was Student’s *t*‐test. Significance was showed by *P > 0.05, **P > 0.01 and ***P > 0.001.

## 3 Results

### 3.1 Induction of TCFL5 expression in human colorectal cancer

The relationship between TCFL5 and CRC was firstly addressed by databases analysis [29, 30, 31, 32, 33]. *TCFL5* was significantly upregulated in carcinoma tissue (Fig. S1A) being higher in advanced tumour stages (Fig. S1B), mostly in tumours that present metastasis in 1–3 lymph nodes and distant metastasis (Fig. S1C–D). Moreover, 212 out of 524 CRC cases (40%) showed some genetic alterations in *TCFL5* (Table S2). The most represented alteration was an increase in TCFL5 mRNA levels (32%), being locus amplification much less abundant (2%). A combination of mRNA high expression/amplification was found in 4% of the cases, mutation/amplification in 1% and single mutations only in 1%.

### 3.2 CRC cell lines express several TCFL5 transcripts

**Fig. 1:**
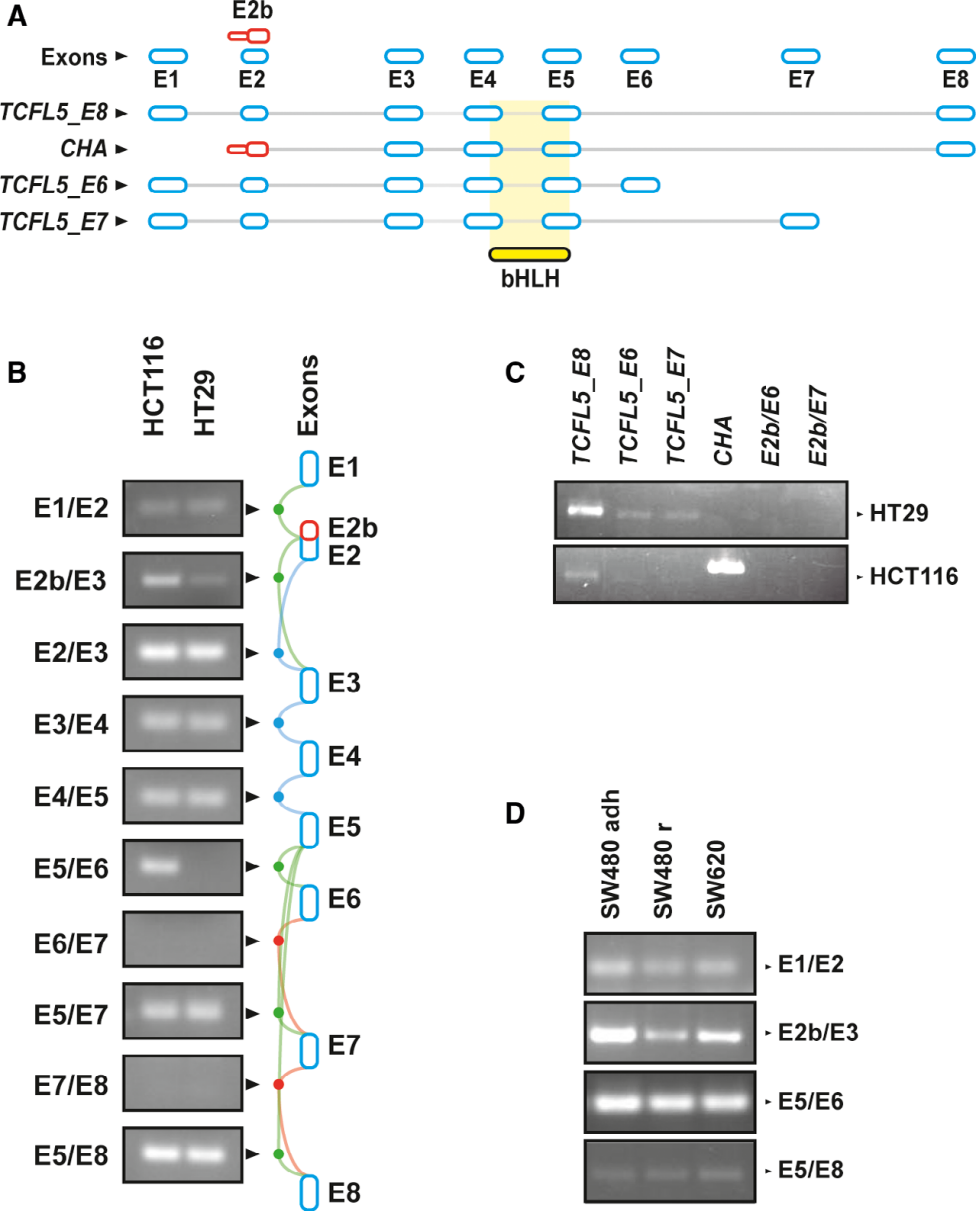
TCFL5 presents several isoforms in CRC cell lines. (A) Scheme of *TCFL5* mRNAs described in databases. In blue, canonical exons. In red, alternative E2b first exon. bHLH domain is marked in yellow. (B) Expression of exon junctions by RT‐PCR in HCT116 and HT29 CRC cell line. To the left, RT‐PCR analysis for E1/E2, E2b/E3, E2/E3, E3/E4, E4/E5, E5/E6, E6/E7, E5/E7, E7/E8 and E5/E8 exon junctions. To the right, exon junctions scheme. Common exon junctions (blue lines), specific exon junctions (green line) and nondescribed exon junctions (red line) are represented. (C) Expression of complete transcripts *TCFL5_E8*, *TCFL5_E7*, *TCFL5_E6*, *CHA*, *E2b_E7* and *E2b_E6* by RT‐PCR in HCT116 and HT29 CRC cell lines. (D) Expression of exon junctions E1/E2, E2b/E3, E5/E6 and E5/E8 by RT‐PCR in SW480 adherent cells (SW480adh), SW480 round cells (SW480r) and SW620 CRC cell line.

The human *TCFL5* locus is composed of 8 exons: the common central exons: E2, E3, E4 and E5; 3 alternative final exons: E6, E7 or E8; and 2 alternative first exons: E1, and E2b and codifies for 4 alternatives predicted transcripts. All isoforms share the bHLH domain (Fig. 1A). However, only two isoforms have been previously experimentally described: the canonical *TCFL5* (hereafter named *TCFL5_E8*), composed by exons E1, E2, E3 E4, E5 and E8; and *TCFL5_CHA* (hereafter named *CHA*), composed by exons E2b, E3, E4, E5 and E8 [11, 17]. During this work, two new transcripts were identified: *TCFL5_E6*, composed by exons E1, E2, E3, E4, E5 and E6; and *TCFL5_E7*, composed by exons E1, E2, E3, E4, E5 and E7.

The existence of predicted exon junctions was tested by RT‐PCR using mRNA from several CRC lines representative of different stages of CRC progression [34]. The common central core E2/E3, E3/E4 and E4/E5, and isoform‐specific exon junctions E1/E2, E5/E7 and E5/E8 were detected in HCT116 and HT29 CRC cell lines (Fig. 1B). Exon junction E5/E6 was only present in HCT116 cells, indicating the expression of *TCFL5_E6* isoform. E2b/E3 exon junction, representative of *CHA* isoform, was found in HCT116 cells but not in HT29. Exon junctions E6/E7 and E7/E8 were not detected, consistently with the idea that these exon junctions are not produced. This was corroborated in HCT116 and HT29 cells by PCR amplification of complete transcripts from all isoforms (Fig. 1C). In SW620 and SW480 cells, high *CHA* and *TCFL5_E8* mRNA expressions were observed (Fig. 1D). Altogether, these results indicate that all 4 *TCFL5* isoforms are expressed in CRC cells but in a cell line‐dependent manner. Thus, high *CHA* and detectable *TCFL5_E8* expressions were found in HCT116 cells, whereas HT29 showed the opposite pattern.

Detailed analysis of *TCFL5* isoforms in RNAseq from CRC data set [35] showed that *CHA, TCFL5_E6* and *TCFL5_E7* were all detectable in human CRC tumours (Fig. S1E). Since E1 is present in *TCFL5_E6*, *TCFL5_E7 and TCFL5_E8* isoforms*, and E8 in CHA and TCFL5_E8,* no sequence is unique to *TCFL5_E8,* impeding its specific detection. Next, a correlation analysis of *TCFL5* expression and some important carcinogenic CRC mutations was performed. The group with the highest expression of *TCFL5* presented more samples with mutant *TP53* and APC (92% of the cases) (Table S3). Moreover, specific E2b (*CHA*) and E1 expression levels were also positively related with mutated *TP53* and *APC*, confirming a correlation between *TCFL5* and *TP53*, and *APC*. In contrast, a negative correlation with *PTEN* and *PIK3CA*‐activating mutations, but not with *K*‐*RAS*, was observed.

### 3.3 TCFL5_E8 and CHA induce an opposite phenotype in CRC cells

**Fig. 2:**
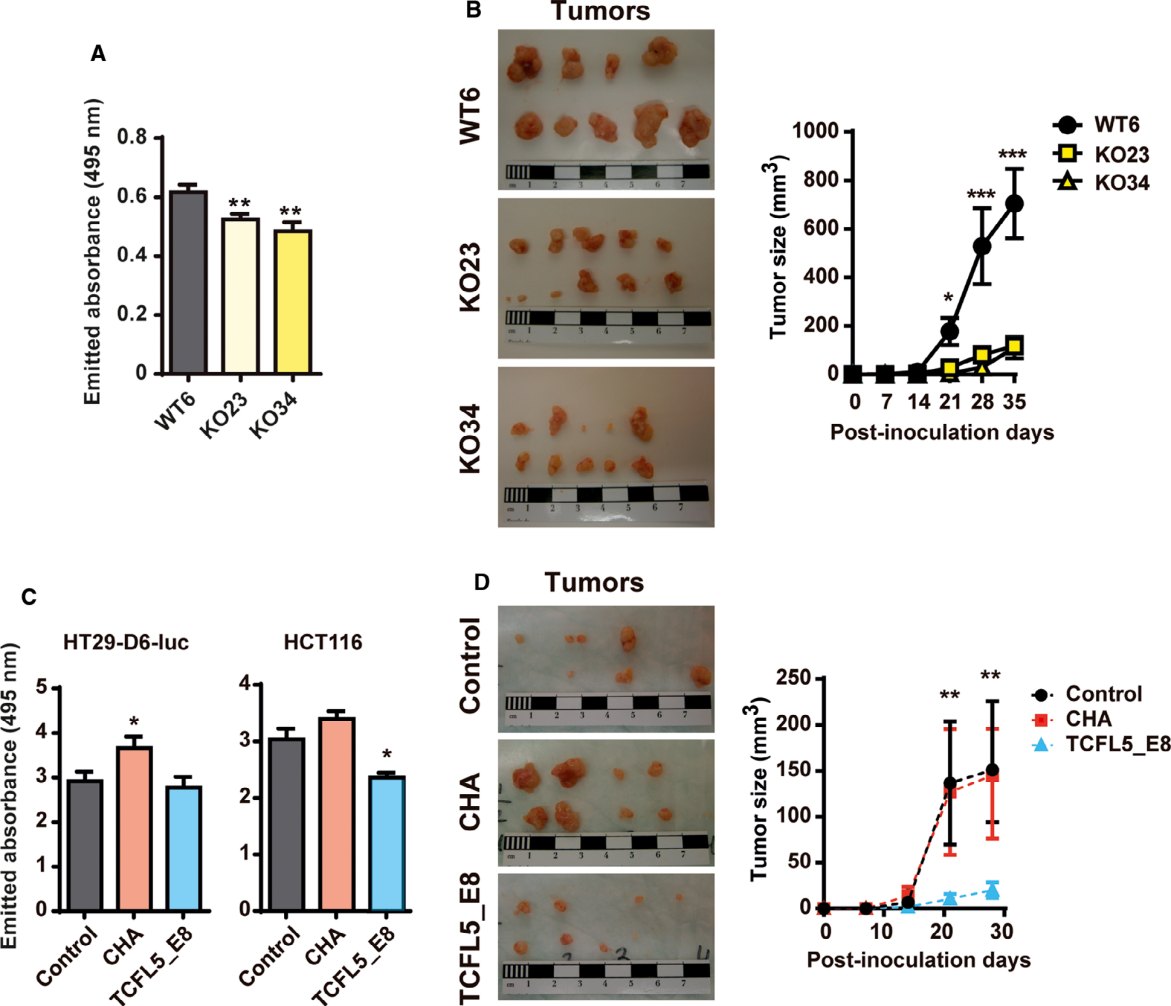
TCFL5 alters proliferation capacity in CRC‐modified cell lines. (A) Proliferation was determined by Alamar Blue assays in WT, KO23 and KO34 HCT116 cell lines. (B) *In vivo* tumour formation of WT (circle), KO23 (square) and KO34 (triangle) HCT116 cell lines. To the left, representative images of the tumour volume on the final day. To the right, volume xenograft tumours were measured for 5 weeks. (C) Proliferation determined by Alamar Blue assay in EV, CHA, and TCFL5_E8 HT29 (left) and HCT116 (right) overexpressing cell lines. (D) *In vivo* tumour formation of CHA (square), and TCFL5_E8 (triangle) overexpressing HCT116 cell lines. To the left, representative images of the tumour volume on the final day. To the right, the tumour volume of xenografts was measured for 4 weeks. Results are expressed as mean ± SEM of three independent experiments (*t*‐test; *P < 0.05, **P < 0.01, ***P < 0.001).

A complete knockout of all TCFL5 isoforms was generated by exon 3 deletion (Fig. S2A‐B). Control HCT116‐WT cells, which maintained exon 3 after CRISPR‐Cas9 transfection, showed similar *TCFL5* levels as the parental cell line. Two clones lacking exon 3, HCT116‐KO23 and HCT116‐KO34, presented a strong reduction in TCFL5_E8 and CHA mRNA and protein expression (Fig. S2C‐E) and exhibited a slower proliferation by either cell counting (Fig. S3A) or Alamar Blue assay (Fig. 2A). Similarly, generated HT29 and SW620 TCFL5 knockout cells were unable to grow (data not shown), suggesting requirement of TCFL5 for cell viability. Thus, shRNAs were used to reduce but not eliminate *TCFL5* in HT29 cell line. This approach resulted in a reduction of cell growth (Fig. S3B). In the same way, the use of specific siRNA against *CHA* in HCT116 and HEK‐293T cells produced a significant decrease in proliferation (Fig. S3C‐D). Finally, evaluating the ability of HCT116‐KOs cells to grow tumours *in vivo*, we confirmed that HCT116‐KOs had significantly lower proliferation capacity than control HCT116‐WT cells (Fig. 2B).

Stable overexpression of CHA and TCFL5_E8 in HCT116 and HT29 cells was obtained (Fig. S2F–I). Surprisingly, each isoform behaves differently depending on the cell line. CHA overexpression produced a significant increase in the proliferation of HT29 but not HCT116 cells. In contrast, TCFL5_E8 overexpression produced a significant decrease in HCT116 cell proliferation, not affecting HT29 cells (Fig. 2C and S3E). The most striking effect was observed *in vivo* while studying the tumorigenic capacity of these cells: CHA overexpression did not affect while TCFL5_E8 overexpression drastically reduced the tumour growth of HCT116 xenografts (Fig. 2D).

Colony formation capacity was not affected in HCT116‐KOs cells (Fig. S3F). However, CHA and TCFL5_E8 overexpression differentially affected colony formation capacity: while CHA overexpression resulted in a significantly higher colony formation capacity in HT29 but not in HCT116 cells, TCFL5_E8 overexpression resulted in fewer colonies in HCT116 but not in HT29 cells (Fig. S3G).

**Fig. 3:**
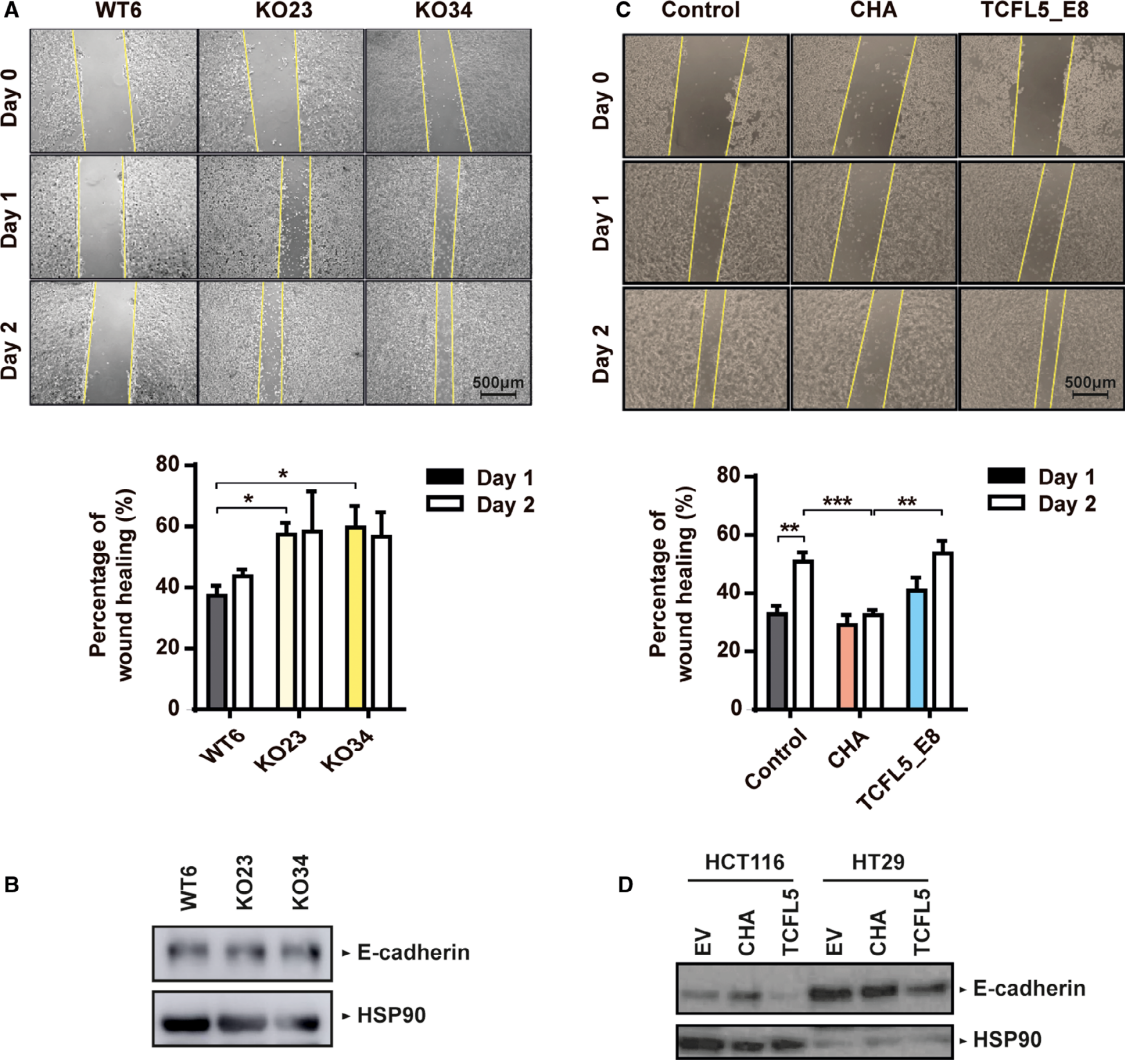
CHA reduces migration capacity in HCT116‐modified cell lines. (A) Migration capacity determined by wound healing assay in WT, KO23, and KO34 HCT116 cell lines at different time points: 1 day (filled bars) and 2 days (empty bars). Above, representative images of wound healing assay. Scale bar 500μm. Below, the percentage of wound healing. (B) Protein levels of E‐cadherin 1 and HSP90 by WB in WT, KO23 and KO34 HCT116 cell lines. (C) Migration capacity determined by wound healing assay in EV, CHA and TCFL5_E8 HCT116 cell lines at different time points: 1 day (filled bars) and 2 days (empty bars). Above, representative images of wound healing assay. Scale bar 500 μm. Below, the percentage of wound healing. (D) Protein levels of E‐cadherin and HSP90 by WB in HCT116 and HT29‐overexpressed cell lines. Results are expressed as mean ± SEM of three independent experiments (*t*‐test; *P < 0.05, **P < 0.01, ***P < 0.001).

Both HCT116‐KO cell lines showed higher migration capacity than HCT116‐WT cells (Fig. 3A), which was not accompanied by any E‐cadherin protein expression change (Fig. 3B). On the other hand, CHA overexpression reduced cell migration while TCFL5_E8 did not affect it (Fig. 3C). Consequently, overexpression of CHA increased E‐cadherin protein expression in HCT116, but not in HT29, while TCFL5_E8 reduced it in both HCT116 and HT29 (Fig. 3D).

### 3.4 TCFL5 isoforms are induced during spheroids formation

**Fig. 4:**
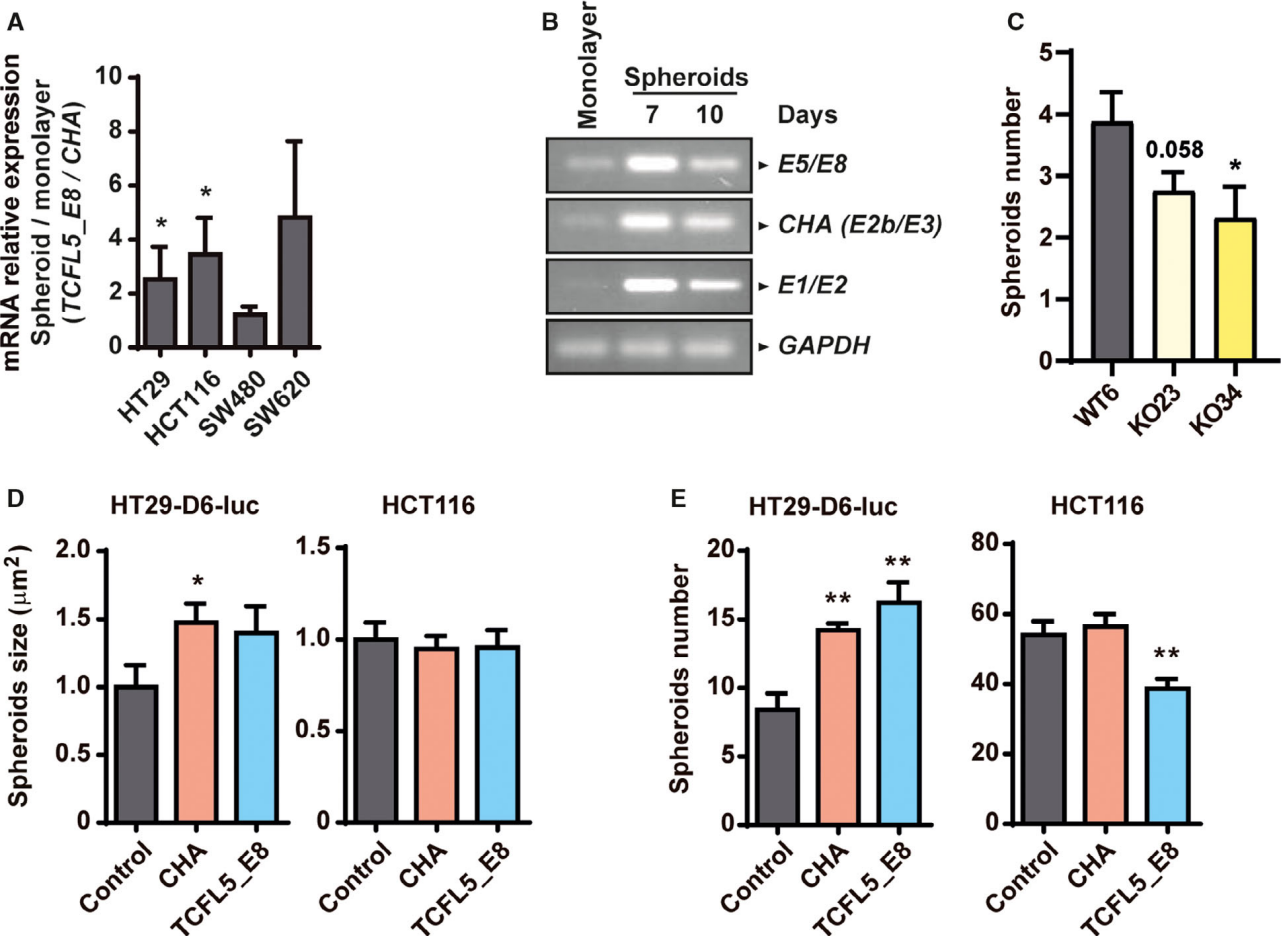
TCFL5 isoforms were induced in spheroids. (A) Relative levels of *TCFL5_E8*/*CHA* mRNA by qPCR in spheroid compared with the monolayer of HCT116, HT29, SW480 and SW620 CRC cell lines. (B) E1/E2, E5/E8 and specific expression of *CHA* (E2b/E3) mRNA by PCR in spheroids compared with the monolayer in HCT116 CRC cell line. (C) Number of spheroids in WT, KO23 and KO34 HCT116‐modified cell lines. (D) Size of spheroids of EV, CHA, and TCFL5_E8 HCT116 and HT29. (E) Number of spheroids of EV, CHA, and TCFL5_E8 HCT116 and HT29. Results are expressed as mean ± SEM of three independent experiments (*t*‐test; *P < 0.05, **P < 0.01).

Previous studies demonstrated induction of *TCFL5* during spheroids formation in HT29 cells [24] but those did not discriminate between TCFL5 isoforms. E5/E8 was higher in spheroids of HCT116 and HT29 as well as in SW620 cells, although not statistically significant in the last case, but not in SW480 cells, comparing with monolayers (Fig. 4A). *TCFL5*_*E8* and *CHA* induction in spheroids, compared with monolayer cultures, was addressed using specific oligonucleotides (Fig. 4B): E2b/E3 (specific for *CHA*), E1/E2 and E5/E8 exon junctions were induced.

Next, we generated spheroids of the established cell lines to elucidate the effect of TCFL5 in this process. HCT116‐KOs cell lines showed a significant reduction in the number of spheroids formed compared with HCT116‐WT cells (Fig. 4C). Again, in overexpressing cells, results were isoform and cell‐line‐dependent. CHA and TCFL5_E8 overexpression produced more and larger spheroids in the HT29. In HCT116, neither isoform affected the size while TCFL5_E8 reduced the number of spheroids formed (Fig. 4D‐E).

### 3.5 CHA and TCFL5_E8 control SOX2 and KLF4 transcription

**Fig. 5:**
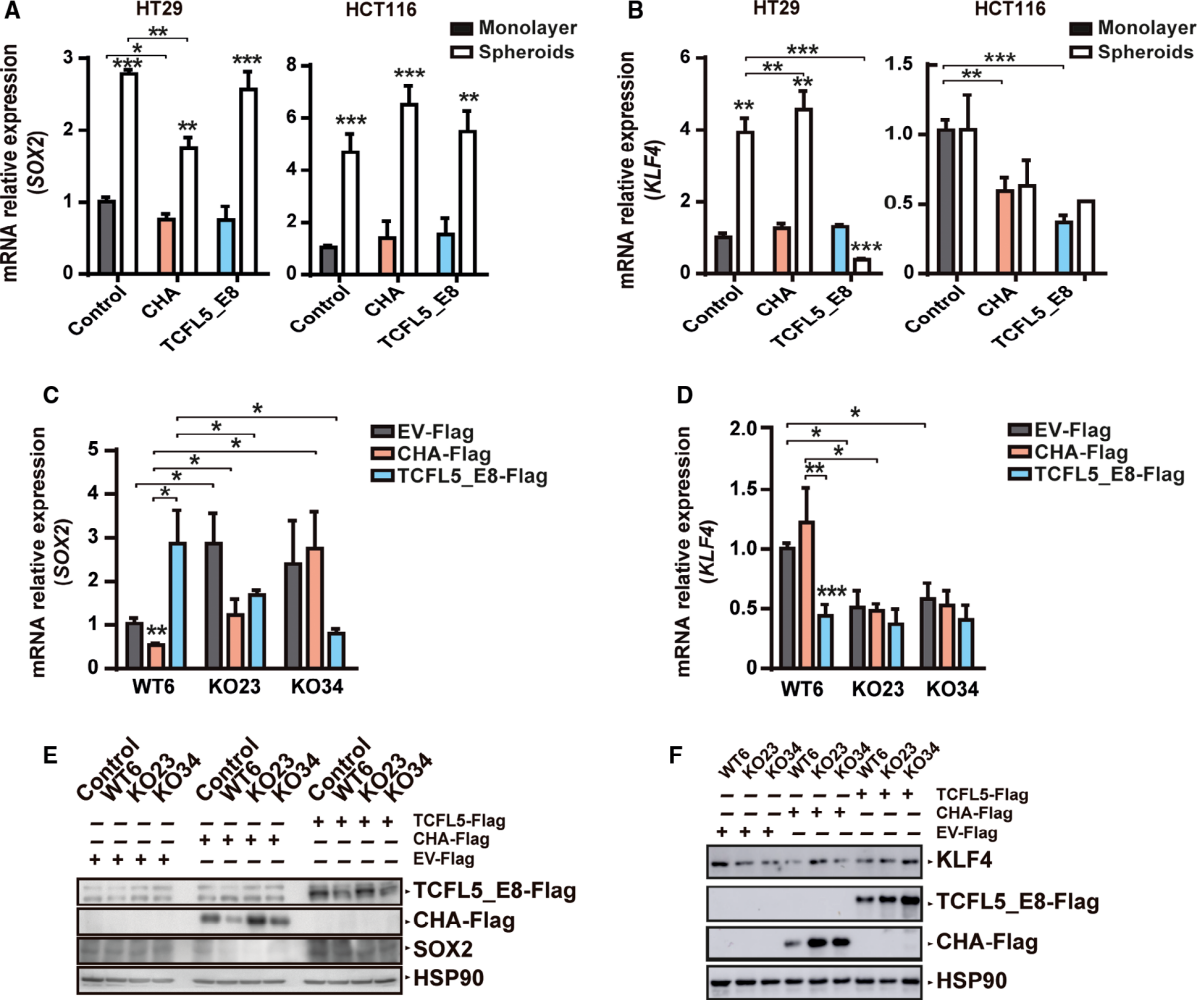
CHA and TCFL5_E8 regulate *SOX2* and *KLF4* transcription. (A) Relative levels of *SOX2* mRNA by RT‐qPCR in spheroids (empty bars) compared with monolayer (filled bars) in EV, CHA, and TCFL5_E8 HT29 (left) and HCT116 (right) cell lines. (B) Relative levels of *KLF4* mRNA by RT‐qPCR in spheroid (empty bars) compared with monolayer (filled bars) in EV, CHA, and TCFL5_E8 HT29 (left) and HCT116 (right) cell lines. (C) Relative levels of *SOX2* mRNA by RT‐qPCR in WT, KO23 and KO34 HCT116 cell lines with control (grey bars), CHA (red bars) or TCFL5_E8 (blue bars) transient expression. (D) Relative levels of *KLF4* mRNA by RT‐qPCR in WT, KO23 and KO34 HCT116 cell lines with control (grey bars), CHA (red bars) or TCFL5_E8 (blue bars) transient expression. (E) Protein levels of CHA, TCFL5_E8, SOX2 and HSP90 by WB in WT, KO23 and KO34 HCT116 cell lines with control, CHA or TCFL5‐E8 transient expression. (F) Protein levels of CHA, TCFL5_E8, KLF4 and HSP90 by WB in WT, KO23 and KO34 HCT116 cell lines with control, CHA or TCFL5‐E8 transient expression. Results are expressed as mean ± SEM of three independent experiments (*t*‐test; *P < 0.05, **P < 0.01, ***P < 0.001).

Spheroids are highly associated with pluripotency capacity and are used as surrogate systems to evaluate the characteristics of CSCs *in vitro* [36, 37]. We wondered whether there was any relationship between *TCFL5_E8/CHA* induction in spheroids and pluripotency marker expression. No difference was found between common CSC markers such as ALDH1, LGR5 or EPHB2 in spheroids formed by CHA or TCFL5_E8 overexpressing cells and those of control cells (data not shown). *SOX2* expression was high in spheroids from HCT116 and HT29 control cells. TCFL5_E8 and CHA overexpression in HCT116 cells showed no significant changes in *SOX2* expression either in monolayer or in spheroids. However, CHA overexpression in HT29 cell line produced a decrease in *SOX2* mRNA levels in monolayer and spheroids (Fig. 5A). *KLF4* mRNA expression was elevated in spheroids from HT29 but not from HCT116. In HT29, CHA overexpression did not produce a significant effect on *KLF4* expression neither in monolayers nor in spheroids while TCFL5_E8 overexpression completely repressed mRNA *KLF4* expression in spheroids. In HCT116 cells, both CHA and TCFL5_E8 reduced *KLF4* levels in monolayer (Fig. 5B).

Our results support the idea that TCFL5_E8/CHA affects *SOX2* and *KLF4* expression in a cell‐line‐dependent manner. This condition could be explained by the basal *TCFL5* locus expression of each cell line. To confirm this hypothesis, we studied *SOX2* and *KLF4* expression in transient transfection, reintroducing TCFL5_E8/CHA in HCT116‐KO cells. *SOX2* expression was high in both HCT116‐KOs cells compared with HCT116‐WT cells (Fig. 5C). CHA overexpression in HCT116‐WT cells reduced *SOX2* levels comparing with control transfection. Moreover, the recovery of CHA expression in HCT116‐KOs cells reduced *SOX2* expression to basal levels. TCFL5_E8 expression in HCT116‐WT cells produced higher levels of *SOX2*. However, its reintroduction in HCT116‐KO cells reduced *SOX2* expression. *KLF4* expression was reduced in both HCT116‐KOs cells compared with HCT116‐WT cells (Fig. 5D). Transient CHA expression in HCT116‐WT cells did not produce changes in *KLF4* mRNA levels while transient TCFL5_E8 expression reduced *KLF4* expression. Surprisingly, neither CHA nor TCFL5_E8 reintroduction affected *KLF4* expression in HCT116‐KOs cells. Results were confirmed also at the protein level (Fig. 5E‐F). We can conclude that SOX2 and KLF4 expression could be under a complex TCFL5_E8/CHA control.

On the other hand, we observed a reduction in *SOX2* mRNA levels, in CHA overexpressing HCT116 cell‐derived tumours, while TCFL5_E8 overexpression tended to increase it (Fig. S4A). CHA produced an increase in KLF4 *mRNA* levels while TCFL5_E8 overexpression did not have any effect (Fig. S4B). Tumours from HCT116‐KOs cells did not present any statistically significant change in *SOX2* expression (Fig. S4C), but they shared a significant reduction in *KLF4* expression (Fig. S4D).

**Fig. 6:**
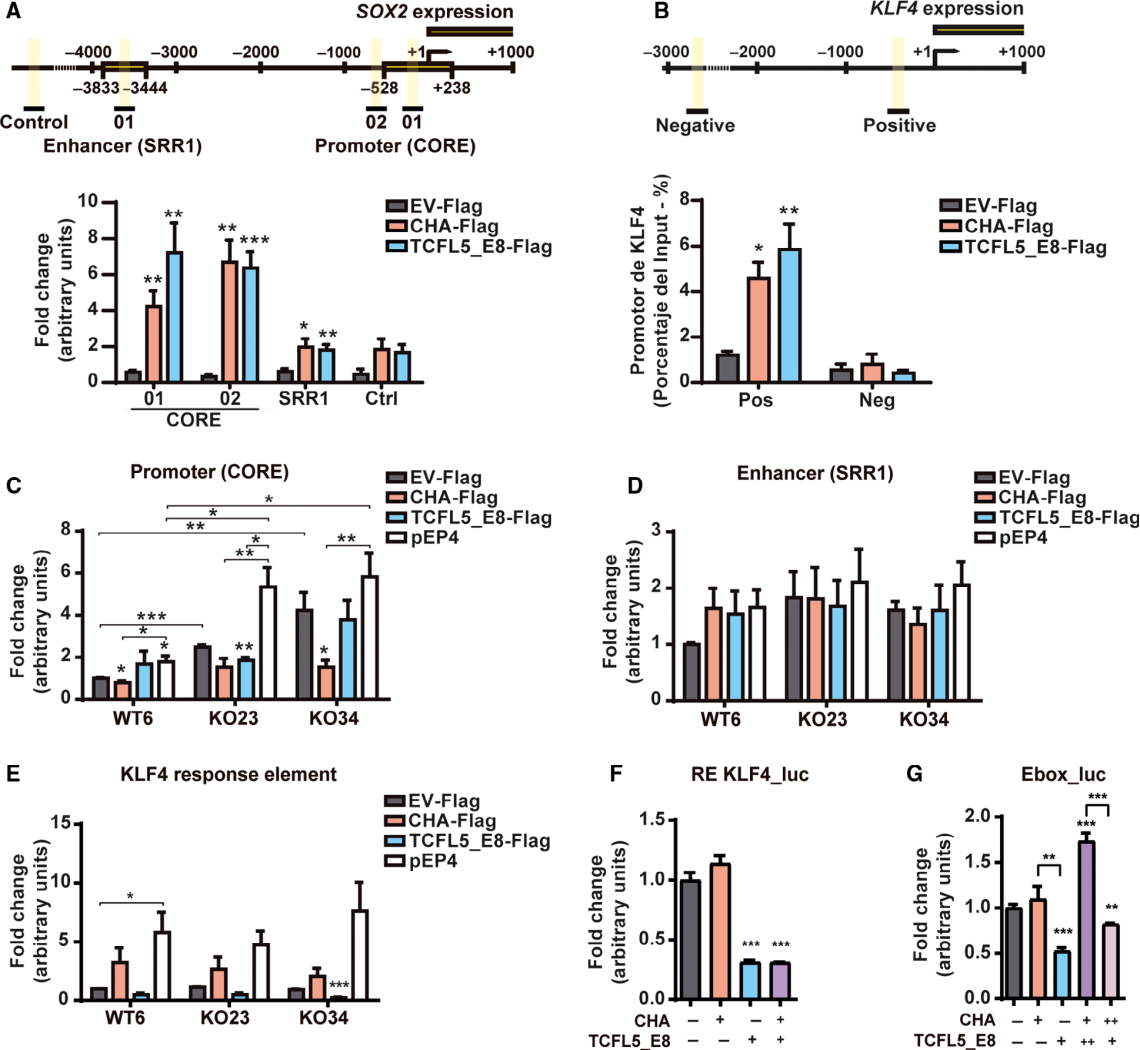
CHA and TCFL5_E8 directly control *SOX2* and *KLF4* promoters. (A) CHA and TCFL5_E8 binding to the *SOX2* promoter. Above, scheme of *SOX2* promoter and region studied by chromatin immunoprecipitation (ChIP). Below, CHA (red bars) and TCFL5_E8 (blue bars) binding to the control, SRR1 and CORE regions of *SOX2* promoter determined by qPCR analysis of immunoprecipitated DNA. (B) CHA and TCFL5_E8 binding to the *KLF4* promoter. Above, scheme of *KLF4* promoter and region studied by ChIP. Below, CHA (red bars) and TCFL5_E8 (blue bars) binding to the negative and positive regions of the *KLF4* promoter determined by qPCR analysis of immunoprecipitated DNA. (C) Luciferase activity of CORE region of *SOX2* promoter in WT, KO23 and KO34 HCT116 cell lines with control (grey bars), CHA (red bars), TCFL5_E8 (blue bars) or pEP4 (white bars) transient expression. (D) Luciferase activity of SRR1 region of *SOX2* promoter in WT, KO23 and KO34 HCT116 cell lines with control (grey bars), CHA (red bars), TCFL5_E8 (blue bars) or pEP4 (white bars) transient expression. (E) KLF4 activity by KLF4‐dependent reporter luciferase assay in WT, KO23 and KO34 HCT116 cell lines with control (grey bars), CHA (red bars), TCFL5_E8 (blue bars) or pEP4 (white grey bars) transient expression. (F) KLF4 activity determined by KLF4‐dependent reporter luciferase assay in HCT116 cells transfected with EV, CHA, TCFL5_E8, and an equal amount of CHA and TCFL5_E8. (G) Luciferase activity of 4× E‐box minimal promoter in HCT116 cells transfected with EV, CHA, TCFL5_E8, and different amounts of CHA and TCFL5_E8. Results are expressed as means ± SEM of three independent experiments (*t*‐test; *P < 0.05, **P < 0.01, ***P < 0.001).

To investigate whether TCFL5_E8 and CHA control over SOX2 and KLF4 expression are due to a direct transcriptional regulation or an indirect mechanism, we studied TCFL5_E8 and CHA binding to *SOX2* and *KLF4* promoters. We analysed two previously described regulatory regions for *SOX2*: CORE and SRR1 [38]. TCFL5_E8 and CHA bound to the CORE region of *SOX2* promoter (Fig. 6A). However, neither TCFL5_E8 nor CHA was able to bind to the SRR1 region. In addition, we found several possible bHLH motifs for TCFL5_E8/CHA in the *SOX2* promoter using the JASPAR database [39]. Indeed, some of them were located in the CORE region, but not in the SRR1 enhancer region (Fig. S4E). Regarding KLF4, we analysed a close (positive) and a faraway (negative) region from the start of transcription. We observed that both TCFL5_E8 and CHA were bound to KLF4 promoter (Fig. 6B). Again, we found bHLH motifs for TCFL5_E8 and CHA in the positive region identified (Fig. S4F).

Then, we studied whether CHA and TCFL5_E8 binding to *SOX2* promoter led to alterations in its activity. The effect of the two isoforms on the control of *SOX2* promoter regions (CORE and SRR1) was analysed by luciferase assays. As a positive control, pEP4 E02S CK2 M EN2L plasmid was used, which induces the activity of SOX2 promoter. CORE promoter activity was higher in HCT116‐KOs cells than in HCT116‐WT. This activity was even higher in the presence of the pEP4 plasmid (Fig. 6C). On the contrary, CHA overexpression reduced CORE promoter activity in HCT116‐WT6, and transient transfection of CHA in HCT116‐KOs cells reduced CORE promoter activity to the same level as HCT116‐WT. On the other hand, transient expression of TCFL5_E8 did not produce an effect on SOX2 promoter activity in any condition. Regarding SRR1 promoter region, both TCFL5 deletion and TCFL5_E8/CHA overexpression did not have a significant effect on its activity (Fig. 6D). In addition, the control of KLF4 by TCFL5_E8 and CHA was confirmed by testing the KLF4 transcriptional activity. KLF4‐dependent reporter activity was not affected in HCT116‐KOs cells. However, while CHA overexpression had no significant effect, TCFL5_E8 reduced KLF4 activity in these cells (Fig. 6E). Moreover, CHA co‐expression with TCFL5_E8 did not revert the effect of TCFL5_E8 (Fig. 6F). These results confirmed that CHA, but not TCFL5, is a negative regulator of SOX2 transcription, while TCFL5_E8 negatively regulates KLF4.

Interestingly, assays done with a luciferase reporter controlled by a bHLH responsive region, with putative TCFL5‐binding sites (15), indicate that TCFL5_E8 is a repressor whereas CHA is not. Moreover, CHA antagonized E8 activity (Fig 6G). Together, this and the previous experiments indicate that there is a clear antagonism of both isoforms.

Finally, a possible correlation of *TCFL5* with *SOX2* or *KLF4* mRNA levels in human CRC was analysed [40]. Positive correlation of *TCFL5* and *SOX2* was found in the normal region adjacent to the tumour samples but not in healthy tissue or tumour samples, while *KLF4* negatively correlated with *TCFL5* in tumour samples (Fig. S5A). Moreover, we compared the expression of each *TCFL5* exon with that of *SOX2* and *KLF4* in tumour samples. No correlation of any *TCFL5* exon with *SOX2* in the tumour region was found (Fig. S5B). However, E2b and E8 presented a greater negative correlation with *KLF4* than E1 suggesting that *KLF4* is more related to *CHA* than to *TCFL5*_E8 (Fig. S5C). E2b and E8 showed a better positive correlation than E1 and E8 suggesting that *CHA* is the most relevant isoform in CRC progression (Fig. S5D).

## 4 Discussion

A few studies have superficially indicated that TCFL5 may have a role in CRC. *TCFL5* mRNA levels are higher in carcinomas than in adenomas, but this has been related to a 20q chromosomal amplification frequent in CRC [21, 41]. However, our analysis of the available databases indicates that a significant percentage (40%) of CRC tumours had some kind of alteration in *TCFL5* but in the great majority not resulting from amplification, nor mutation rather from an increase in mRNA levels. These results suggest that higher *TCFL5* levels in CRC tumours may be achieved through transcriptional regulation by the main signalling pathways that control CRC generation such as NOTCH. In this regard, TCFL5 is a direct transcriptional target of NOTCH1 [14] and TCFL5 transcription was confirmed to be activated by NOTCH1 (unpublished Gutierrez‐Nogues A.). Moreover, its higher expression is significantly associated with advanced stages of the malignancy and metastasis.

Previously, only two TCFL5 isoforms (TCFL5_E8 and CHA) were described. However, we demonstrated here that TCFL5 locus might give rise to four different transcripts in CRC cell lines and tumours with different relative expression. Strong evidence was provided that *TCFL5* locus plays a previously underestimated role in CRC and CRC cell line phenotype. This is supported not only by the higher expression in CRC tumours and its correlation with more advanced tumour phenotype but also by our experiments. Thus, knocking down the *TCFL5* locus drastically reduced HCT116 tumour growth ability *in vivo*, proving its relevant role in this process. Moreover, TCFL5‐deficient HCT116 cells show reduced proliferation and higher migration, and a lower spheroid formation capacity. Striking differences between *in vivo* and *in vitro* proliferation could be explained by attending to other processes such as a worse adaptability after inoculation, producing a delay in the tumour proliferation. Noteworthy, generation of complete TCFL5 knockout in other CRC cell lines as HT29 and SW620 were impossible to achieve since this deletion resulted in a loss of cell viability, indirectly indicating a fundamental role of *TCFL5* in controlling survival or proliferation. The reason for those differences is unknown but can rely on differential properties between these cell lines. It is well known that different CRC cell lines represent different types/stages of CRC tumours [32]. An intriguing possibility is that differences can be related to the presence of mutated tumour suppressor genes; thus, HCT116 cells have WT *TP53* and *APC* whereas SW480, SW620 and HT29 have these genes mutated. This possibility fits with the fact that TCFL5 isoforms are significantly more expressed in patient’s tumours that also have those genes mutated than in those with *PTEN* or *PI3KCA* mutations. Thus, the deletion of the complete knockout of *TCFL5* in these cell lines may lead to some deleterious DNA alterations that will require the tumour suppressor activity to maintain cell viability.

Moreover, the complex *TCFL5* locus deficiency effects could be explained by the strikingly different and somewhat opposite behaviours of TCFL5_E8 and CHA isoforms on cellular phenotype. HCT116‐KO cells have reduced proliferation and tumour growth *in vivo,* which is similar to overexpressing TCFL5_E8 in these cells. On the other hand, CHA showed the opposite effect on proliferation, migration and spheroids formation compared with HCT116‐KO cells. A similar inverse relationship was found in migration and E‐cadherin expression where TCFL5_E8 reduced E‐cadherin and CHA induced it.

Previous studies have demonstrated the induction of *TCFL5* in HT29 cells spheroids [24] but did not discriminate between TCFL5 isoforms. Here, we found that *CHA* and *TCFL5*_E8 increased during spheroids formation in both HCT116 and HT29 cells. Spheroid cell cultures are useful for studying cancer development and the basic properties of CSCs. We have demonstrated that TCFL5 KO cells may increase SOX2 and reduce KLF4. SOX2 expression is related to an increment in CSCs and a poor prognosis, promoting an increase in migration and invasion [42, 43, 44]. On the other hand, KLF4 is considered a tumour suppressor gene [45, 46]. CHA and TCFL5_E8 regulate SOX2 and KLF4 expression by binding directly to their promoters in a region where an E‐box is present, but their effect is different: CHA reduces SOX2 resulting in reduced migration, while TCFL5_E8 increases SOX2 and reduces KLF4. Our results with bHLH and KLF4 reporters indicate that there is some antagonism between both isoforms that deserve further experimentation. Interestingly, our analysis of different exons expression in CRC demonstrates that E2b is the exon that mostly correlates with decreasing levels of *KLF4* in CRC samples. In agreement with this, KLF4 knockdown produces an induction of epithelial–mesenchymal transition (EMT) and migration [47], suggesting that the differential effects of TCFL5_E8 and CHA on migration could be mediated through opposite KLF4 regulation. Also, both overexpression and silencing of SOX2 can reduce cell proliferation of CRC cell lines [48]. These observations are in accordance with our data suggesting that proliferation and migration are two non‐necessarily‐dependent processes.

**Fig. 7:**
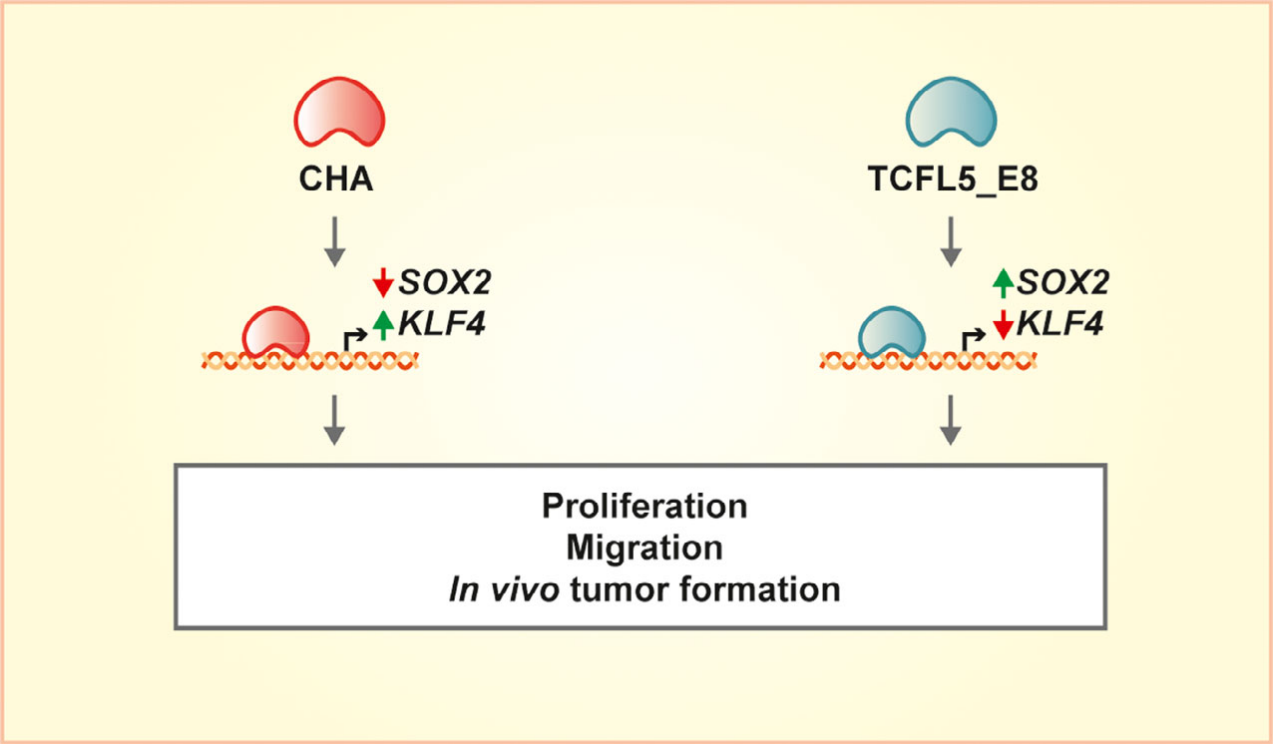
TCFL5_E8 and CHA‐proposed model. TCFL5_E8 and CHA bind to *SOX2* and *KLF4* promoters controlling differently their expression. These effects produce somehow changes in proliferation, migration and tumour formation capacity of the cells depending on the expression of TFL5_E8 or CHA.

TCFL5_E8 and CHA overexpression effects are also CRC cell line‐dependent, which can be explained by the different basal isoform expressions in each line. Thus, HTC116 expresses *CHA*, but little *TCFL5*_*E8*, whereas HT29 expresses *TCFL5*_*E8* but low levels of *CHA*. This expression pattern is consistent with the observed fact that in HT29 cells that have a high basal expression of TCFL5_E8, only CHA overexpression induced an increase in proliferation and spheroids formation. Whereas for HCT116, where CHA basal levels are higher than TCFL5_E8, only TCFL5_E8 overexpression caused a reduction in the carcinogenic traits. Together, our results suggest that it is the TCFL5_E8/CHA ratio that controls the phenotype of the cells. According to this hypothesis, a model can be proposed: higher CHA levels lead to a more aggressive cell phenotype (Fig. 7). Nonetheless, this ratio does not explain completely TCFL5 locus deficiency effects. TCFL5_E6 and TCFL5_E7 isoforms are also expressed in CRC cells so we cannot dismiss the effect of these isoforms on CRC cancer.

The reason for such different functions of TCFL5_E8 and CHA is not understood yet at the molecular level but may be related to being one isoform a dominant inhibitor. The fact that both isoforms can bind to *KLF4* and *SOX2* promoters resulting in opposite effects on their transcription would be in agreement with such hypothesis. The only binding motif described in TCFL5 is the bHLH motif, which is present in both isoforms. They differ only in the presence or absence of the first exon. Thus, exon 1 could confer different properties due to an unknown motif in this region or a different protein structure, thus, promoting different protein interactions. In addition, having opposite roles related to tumoural properties suggests that their expression pattern could be different and controlled independently.

## 5 Conclusions

In summary, our results indicate that TCFL5 plays a more important role than previously thought in CRC controlling SOX2 and KLF4 transcription. This may be due to the existence of several isoforms, of which, the 2 most important play different, and in many circumstances, opposite roles. This may have been overlooked in the analysis of cancer data sets that consider TCFL5 as single mRNA, producing contradictory results or difficult to explain, since the expression of one isoform has a different effect than the other and a very similar phenotype to the one obtained by the complete deletion of the gene.

## Conflict of interest

The authors declare no conflict of interest.

### Peer Review

The peer review history for this article is available at https://publons.com/publon/10.1002/1878‐0261.13085.

## Author contributions

JG‐M, KS, IS‐G and SV‐C performed the experiments and analysed the data. JG‐M, KS, NG and MF designed the study. JG‐M, KS, NG and MF wrote the manuscript. All authors discussed the results and commented on the manuscript.

## Supporting information


**Fig. S1**. TCFL5 expression is higher in human CRC than in normal tissue.

**Fig. S2**. Stable HCT116 and HT29 modified cell lines.

**Fig. S3**. TCFL5 affects proliferation capacity and colony formation of CRC cell lines.

**Fig. S4**. TCFL5_E8 and CHA overexpressed xenografted tumors present alteration in SOX2 and KLF4 expression.

**Fig. S5**. *TCFL5* gene expression correlates with *SOX2* and *KLF4* in human colorectal cancer.

**Fig. S6**. Complete membranes of Western‐blot.

**Table S1**. Sequences of oligonucleotides. Sequences of restriction enzymes or CRISPR guides are underlined.
**Table S2**. TCFL5 gene alteration. TCFL5 alterations were found in the PanCancer Atlas dataset.
**Table S3**. *TCFL5* expression correlates with *TP53* and *APC* mutations. Gene/exon expression, mutation, and clinicopathological data from the TCGA Colon Cancer (COAD) collection were extracted using the UCSC Xena Browser analysis web tool.

## Data Availability

The data that support the findings of this study are available from the corresponding author upon reasonable request.
