# Supplementary figures and images for: Isoform‐specific effects of transcription factor TCFL5 on the pluripotency‐related genes SOX2 and KLF4 in colorectal cancer development
Source: Mol Oncol. 2021 Oct 8;16(9):1876–90. doi: 10.1002/1878-0261.13085 (PMC9067154; doi:10.1002/1878-0261.13085)

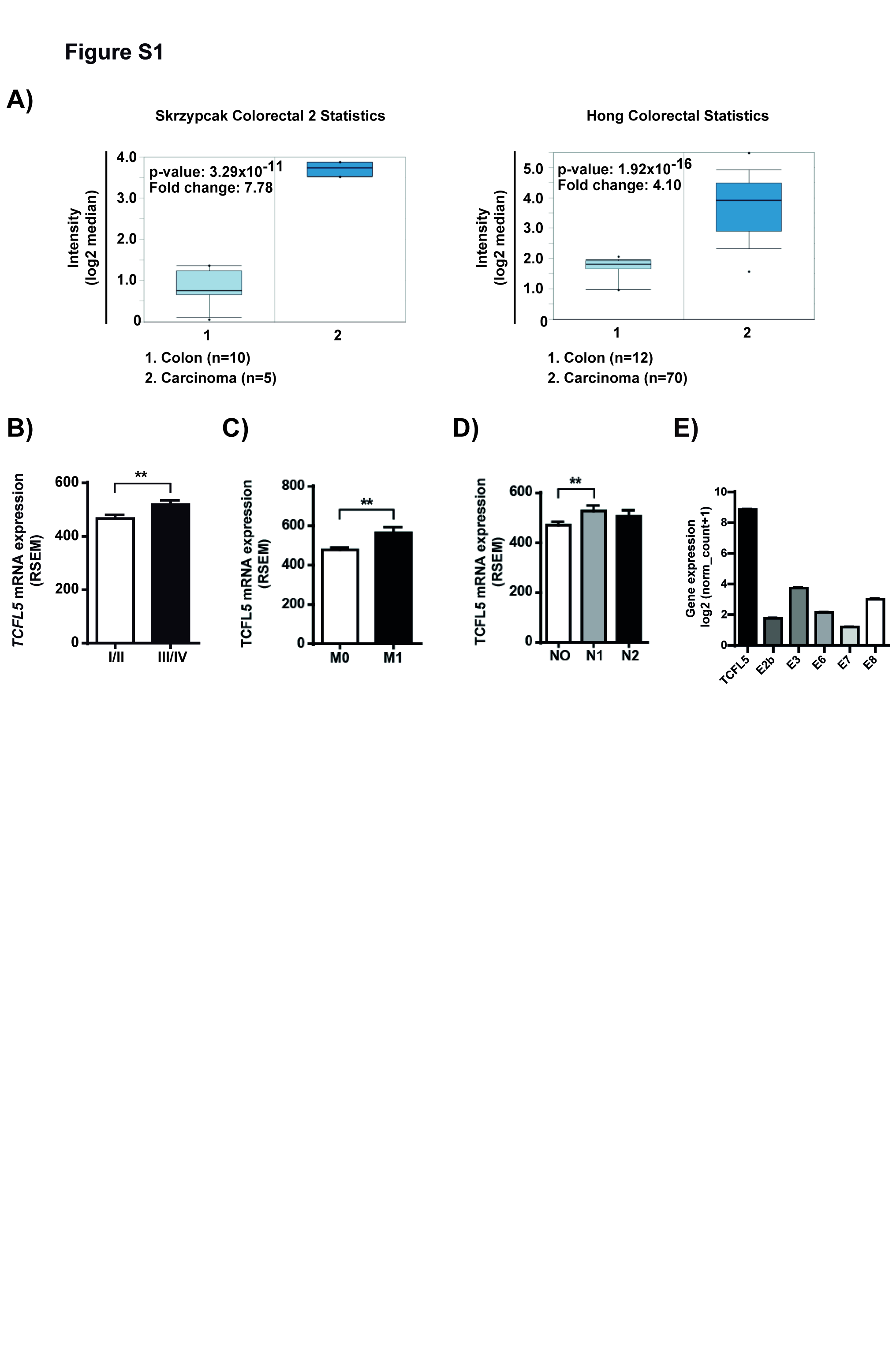

Supplement: Supplementary file 1 — Fig. S1. TCFL5 expression is higher in human CRC than in normal tissue. [file MOL2-16-1876-s002.tif]

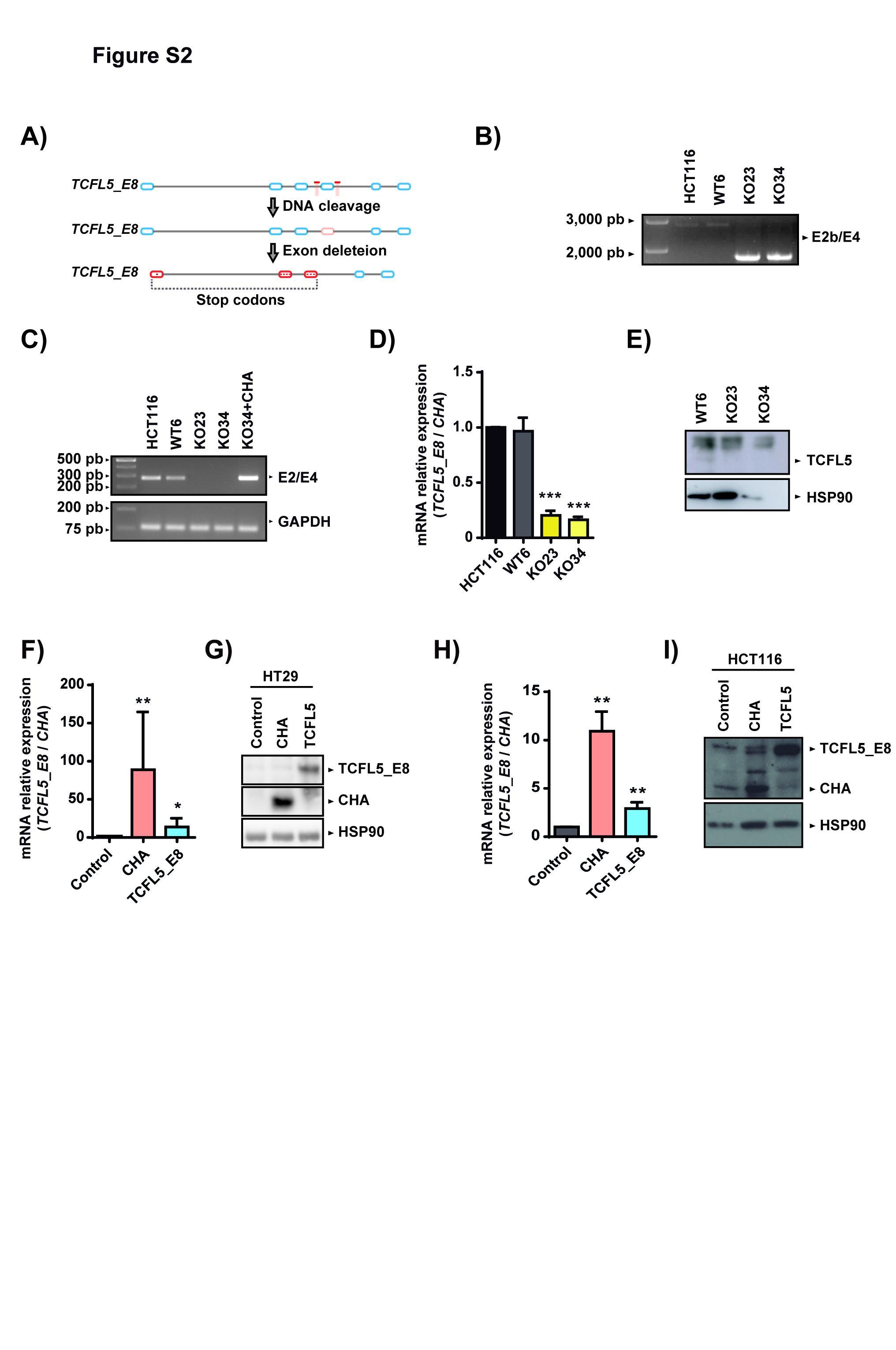

Supplement: Supplementary file 2 — Fig. S2. Stable HCT116 and HT29 modified cell lines. [file MOL2-16-1876-s006.tif]

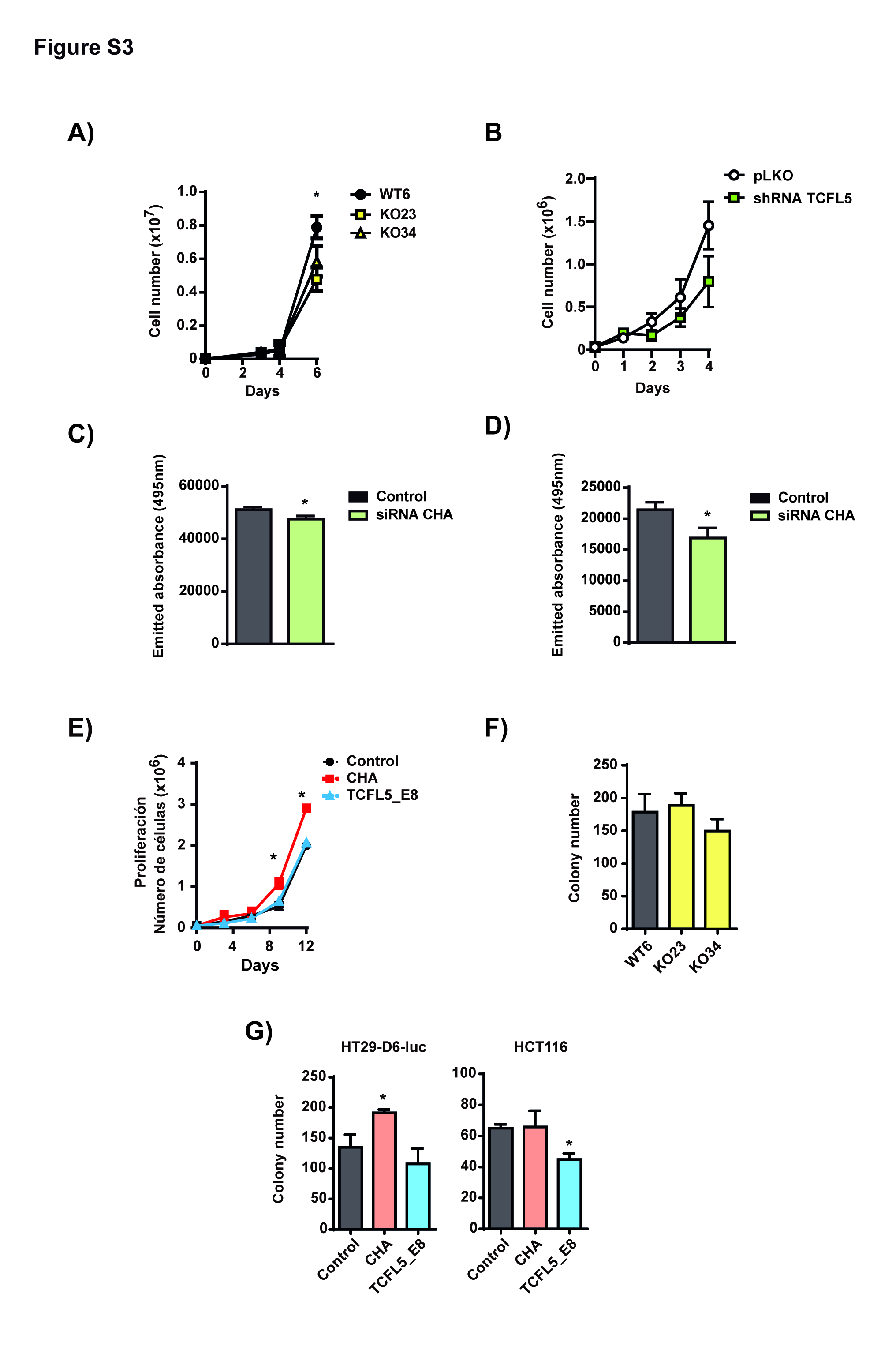

Supplement: Supplementary file 3 — Fig. S3. TCFL5 affects proliferation capacity and colony formation of CRC cell lines. [file MOL2-16-1876-s001.tif]

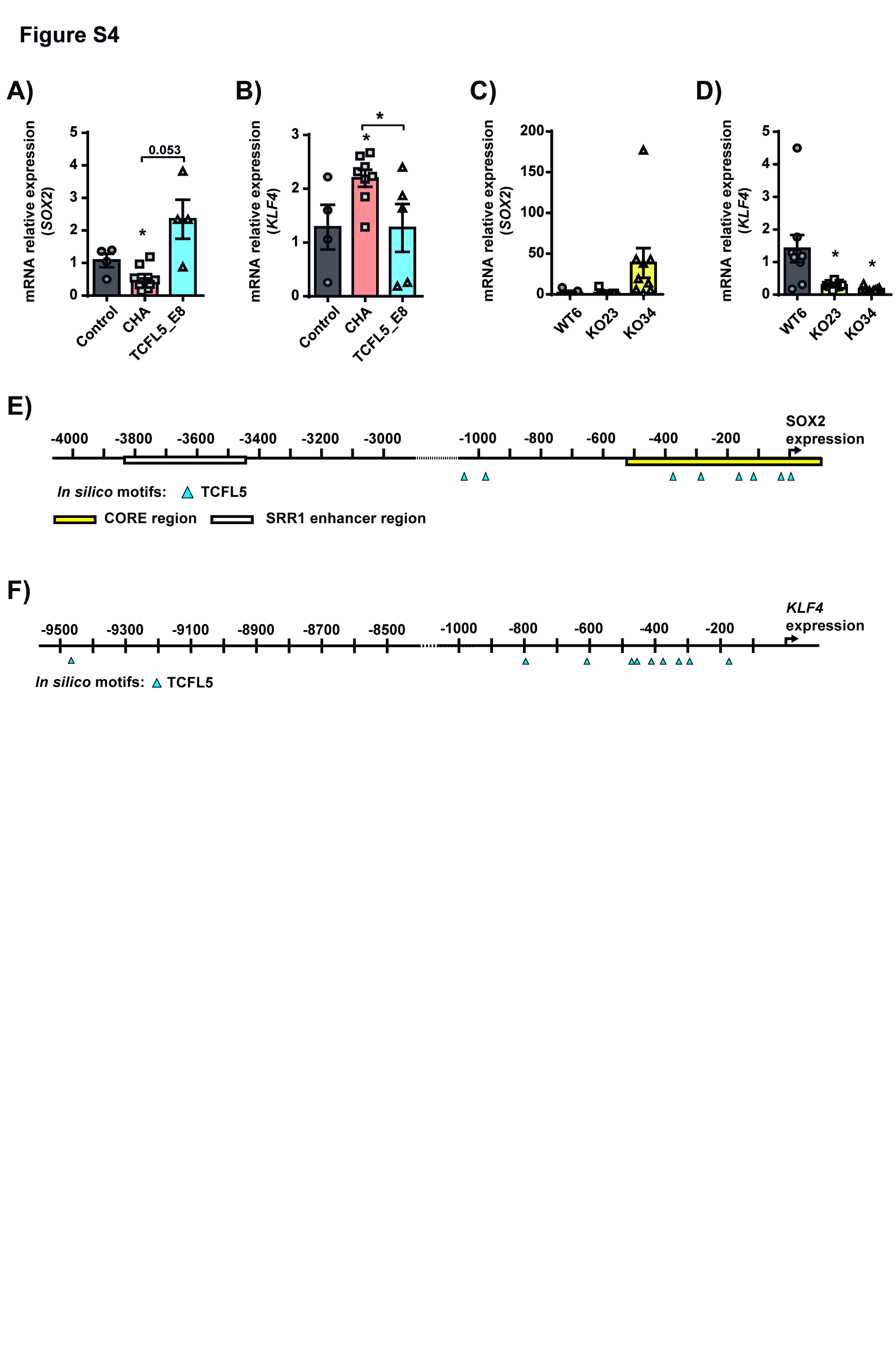

Supplement: Supplementary file 4 — Fig. S4. TCFL5_E8 and CHA overexpressed xenografted tumors present alteration in SOX2 and KLF4 expression. [file MOL2-16-1876-s003.tif]

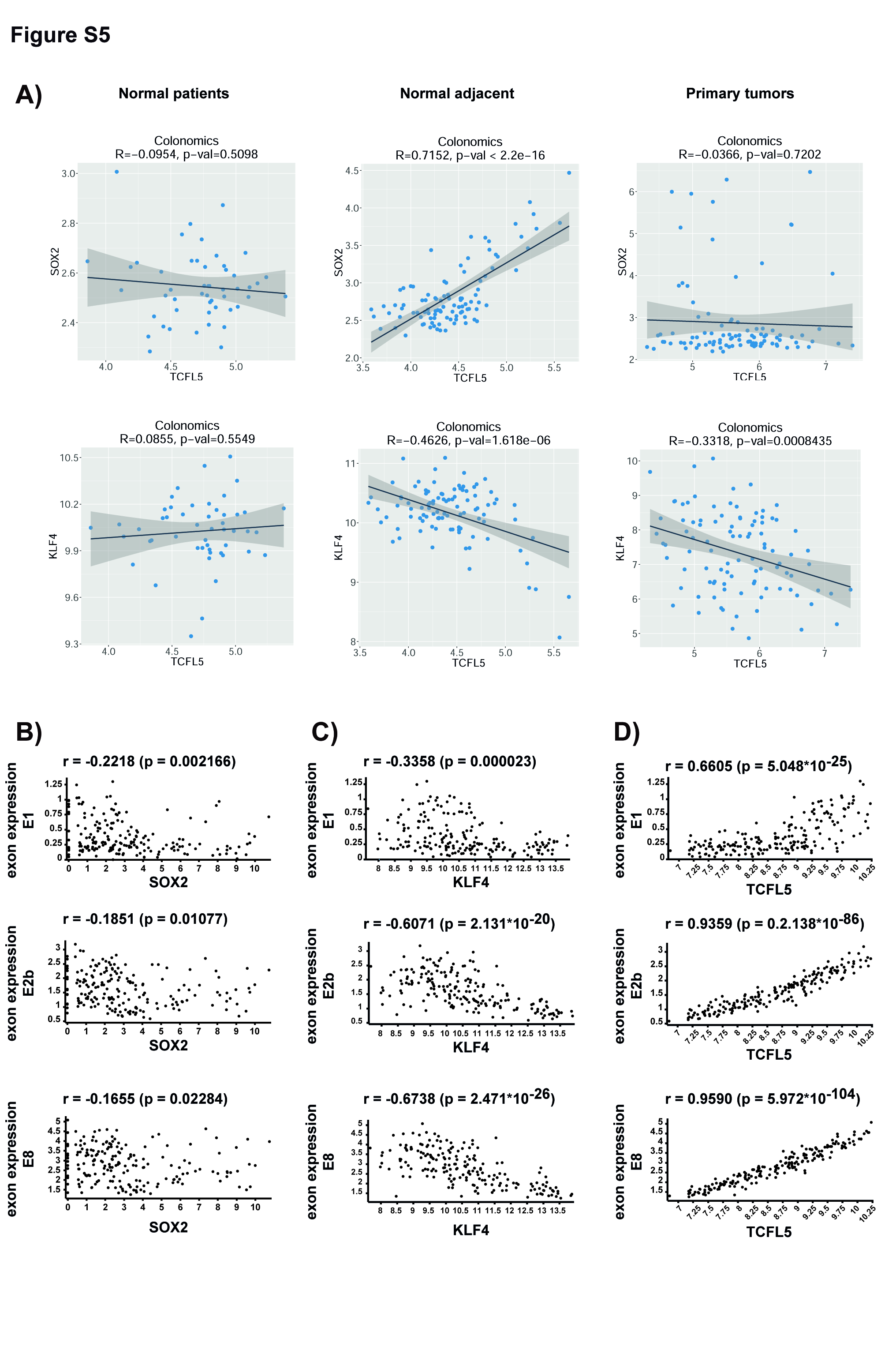

Supplement: Supplementary file 5 — Fig. S5. TCFL5 gene expression correlates with SOX2 and KLF4 in human colorectal cancer. [file MOL2-16-1876-s007.tif]

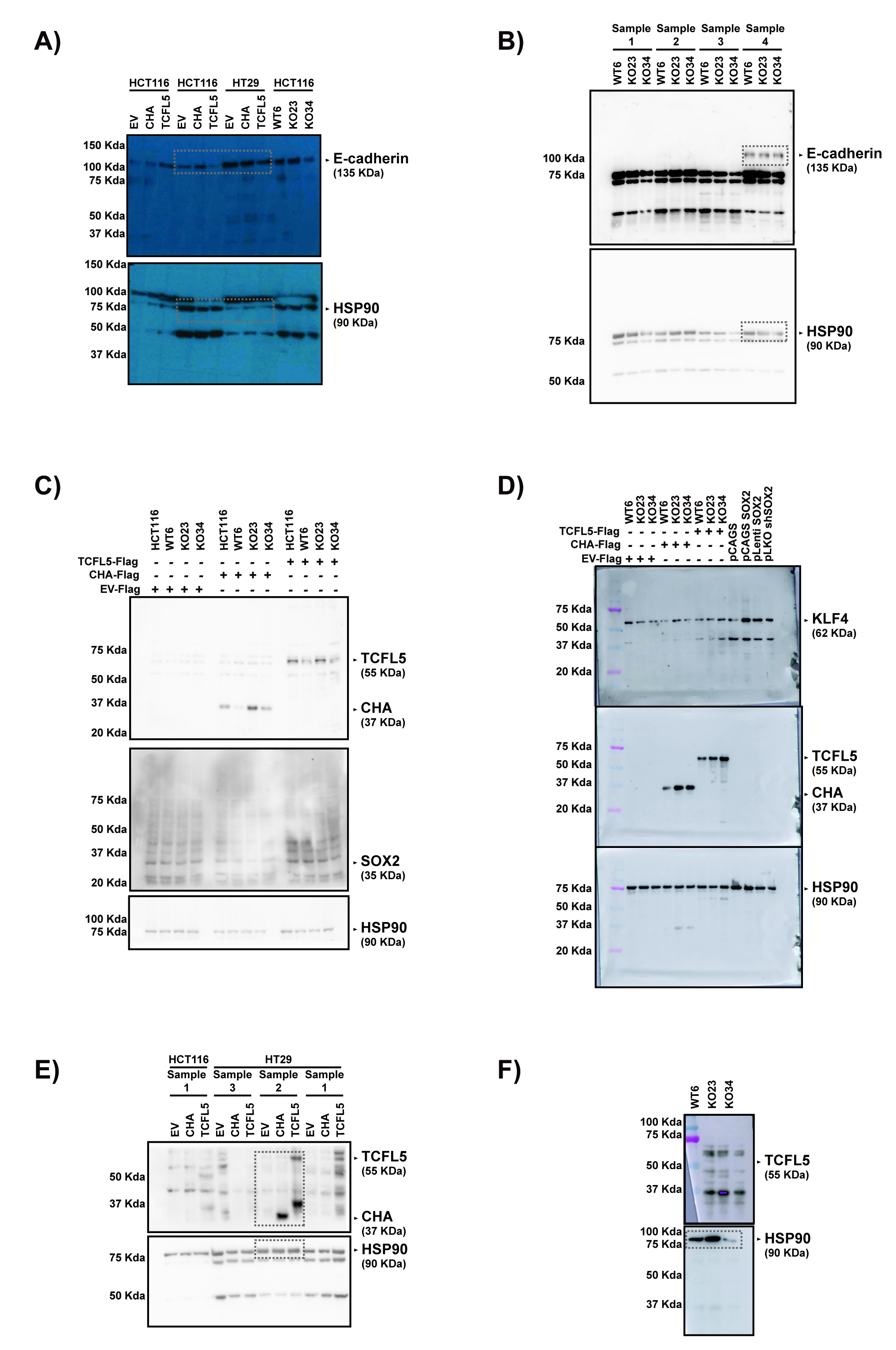

Supplement: Supplementary file 6 — Fig. S6. Complete membranes of Western‐blot. [file MOL2-16-1876-s005.tif]
