# Supplementary material for: Isoform‐specific effects of transcription factor TCFL5 on the pluripotency‐related genes SOX2 and KLF4 in colorectal cancer development
Source: Mol Oncol. 2021 Oct 8;16(9):1876–90. doi: 10.1002/1878-0261.13085 (PMC9067154; doi:10.1002/1878-0261.13085)
Supplement: Supplementary file 7 — Table S1. Sequences of oligonucleotides. Sequences of restriction enzymes or CRISPR guides are underlined. Table S2. TCFL5 gene alteration. TCFL5 alterations were found in the PanCancer Atlas dataset. Table S3. TCFL5 expression correlates with TP53 and APC mutations. Gene/exon expression, mutation, and clinicopathological data from the TCGA Colon Cancer (COAD) collection were extracted using the UCSC Xena Browser analysis web tool. [file MOL2-16-1876-s004.pdf]

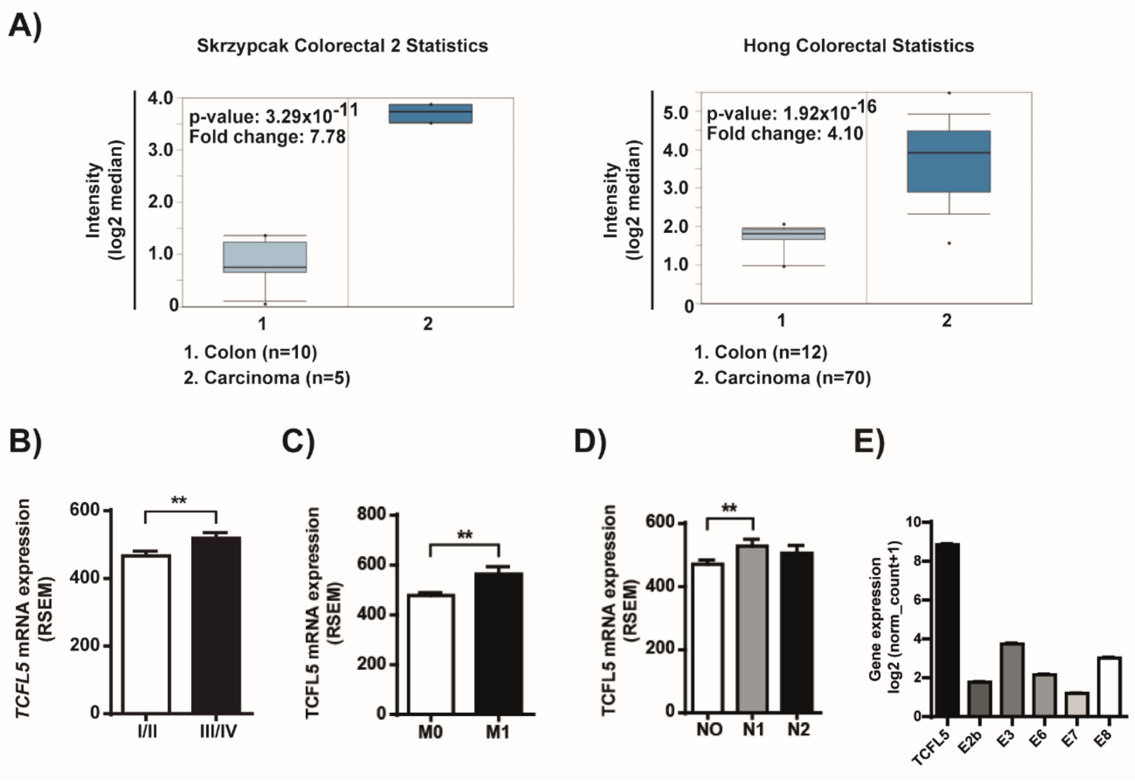

Figure S1. ***TCFL5* expression is higher in human CRC than in normal tissue. A)** *TCFL5* expression in two independent microarrays of human CRC obtained from OncoMIne database: Skyzypack colorectal 2 datasets (left) and Hong colorectal dataset (right). The probe used was 204849_at. **B)** *TCFL5* expression at different stages of human CRC progression. TCGA PanCancer Atlas project dataset was obtained from cBioportal database. **C)** *TCFL5* expression in samples without distant metastases (M0) and with distant metastases (M1). **D)** *TCFL5* expression in samples with no regional lymph node metastasis (N0), metastasis in 1-3 lymph nodes (N1), and metastasis in more than 3 lymph nodes (N2). **E)** Specific *TCFL5* gene or exon expression data in colorectal cancer. Gene/exon expression data from the TCGA Colon Cancer (COAD) collection were extracted using the UCSC Xena Browser analysis web tool. Results are expressed as mean ± SEM (t-test; $^{**}p < 0.01$).

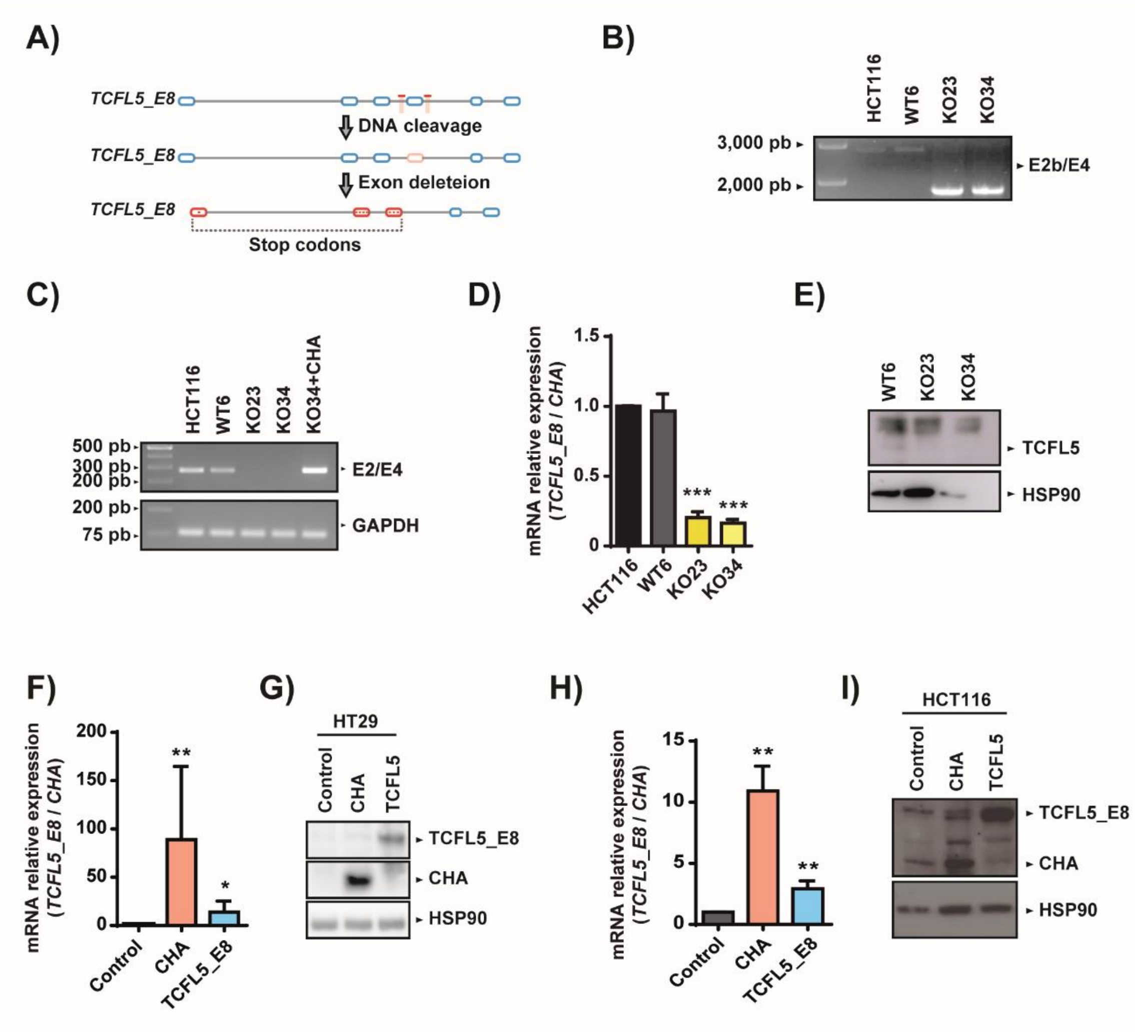

Figure S2. Stable HCT116 and HT29 modified cell lines. A) Scheme for self-cloning CRISPR Cas9 strategy. **B)** Exon E3 deletion in KO23 and KO34 HCT116 cell lines determined by E2b/E4 DNA amplification. WT amplicon is 2885 bp while knock-out amplicon is 1959 bp. **C)** Expression of E2/E4 mRNA by PCR in HCT116 and HCT116 modified cell lines. Transient recovery expression of CHA is showed. **D)** Relative levels of *TCFL5_E8/CHA* mRNA by RT-qPCR in HCT116 and HCT116 modified cell lines. **E)** Protein expression of TCFL5, and HSP90 by WB in WT6, KO23, and KO34 HCT116 cell lines. **F)** Relative levels of *TCFL5_E8/CHA* mRNA by RT-qPCR in EV, CHA, and TCFL5_E8 overexpressing HCT116 cell lines. **G)** Protein expression of TCFL5_E8, CHA, and HSP90 by WB in EV, CHA, and TCFL5_E8 overexpressing HT29 cell lines. **H)** Relative levels of *TCFL5_E8/CHA* mRNA by RT-qPCR EV, CHA, and TCFL5_E8 overexpressing HT29 cell lines. **I)** Protein expression of TCFL5_E8, CHA, and HSP90 by WB in EV, CHA, and TCFL5_E8

overexpressing HCT116 cell lines. Results are expressed as means ± SEM of three independent experiments (t-test; *$p < 0.05$, **$p < 0.01$, ***$p<0.001$).

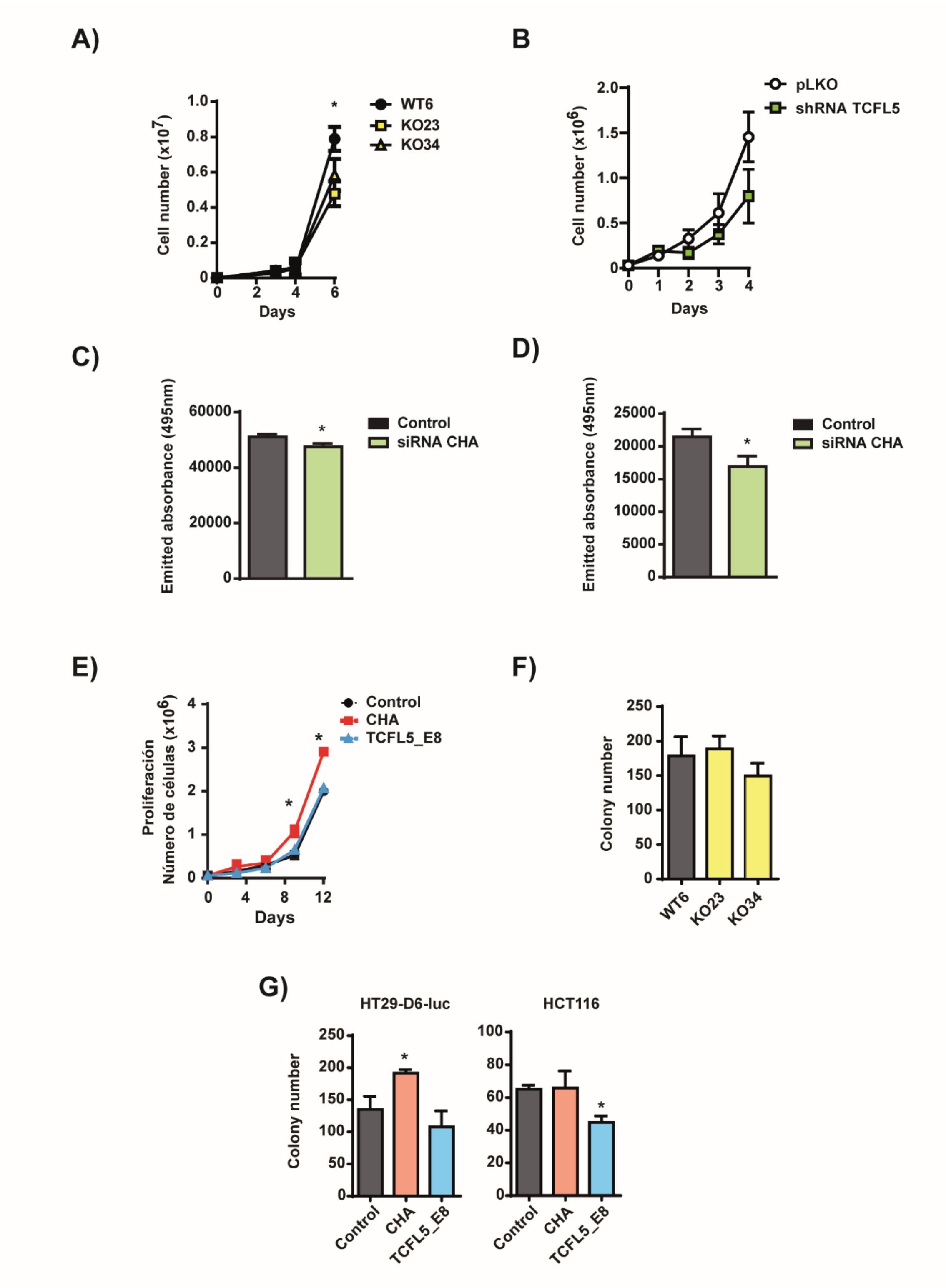

Figure S3. TCFL5 affects proliferation capacity and colony formation of CRC cell lines. A) Proliferation was determined by cell counting at different time points in WT (circle), KO23 (square), and KO34 (triangle) HCT116 cell lines. **B)** Proliferation was determined by cell counting at different time points in pLKO (circle) and shRNA TCFL5 (square) HT29 cell lines. **C)** Proliferation

was determined by Alamar Blue assays after siRNA_scramble and siRNA_CHA transient transfection in HCT116 cell lines. **D)** Proliferation was determined by Alamar Blue assays after siRNA_scramble and siRNA_CHA transient transfection in HEK293T cell lines. **E)** Proliferation was determined by cell counting at different time points in control (circle), CHA (square), and TCFL5_E8 (triangle) HT29 cell lines. **F)** WT, KO23, and KO34 HCT116 cell lines colony capacity determined by the total number of colonies formed at 15 days. **G)** EV, CHA, and TCFL5_E8 overexpressed cell lines colony capacity determined by the total number of colonies formed at 15 days. To the left, HT29 cell lines. To the right, HCT116 cell lines. Results are expressed as means ± SEM of three independent experiments (t-test; $*p < 0.05$, $**p < 0.01$, $***p<0.001$).

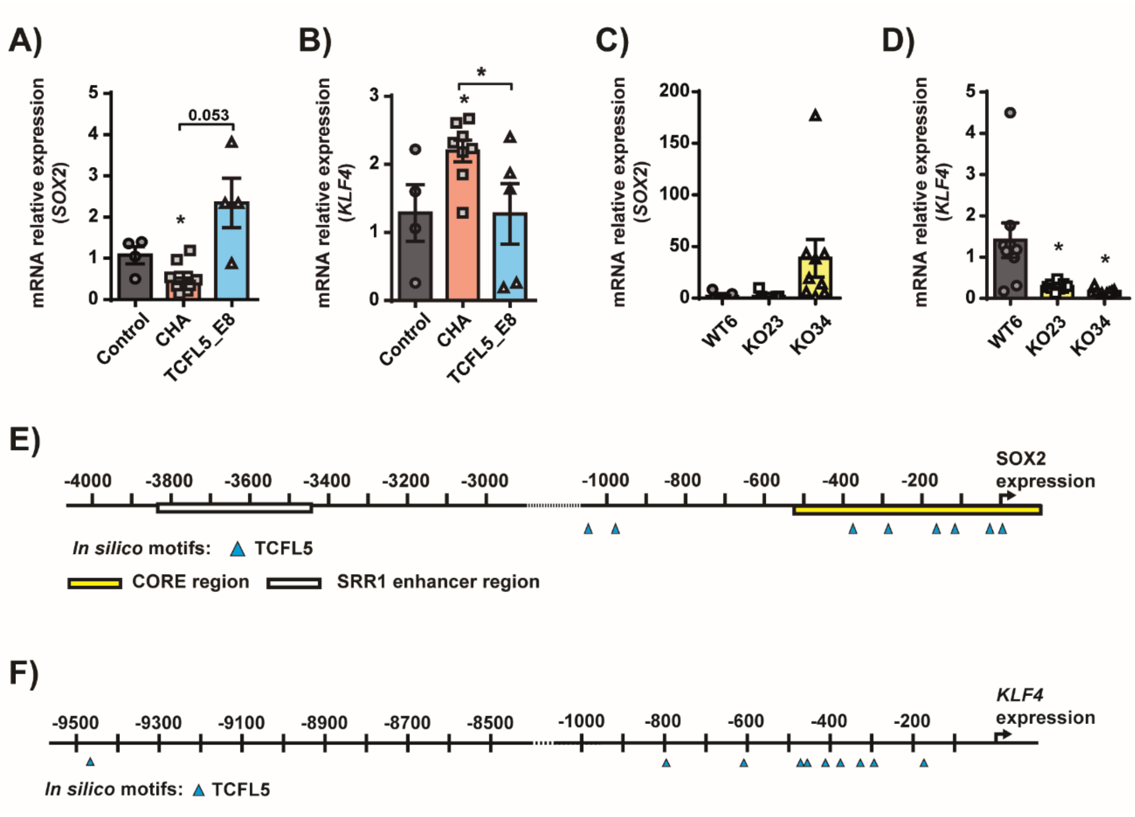

Figure S4. TCFL5_E8 and CHA overexpressed xenografted tumors present alteration in *SOX2* and *KLF4* expression. A) Relative levels of *SOX2* mRNA by RT-qPCR in the xenografted tumors of HCT116 overexpressed cell lines. **B)** Relative levels of *KLF4* mRNA by RT-qPCR in the xenografted tumors of HCT116 overexpressed cell lines. **C)** Relative levels of *SOX2* mRNA by RT-qPCR in the xenografted tumors of WT, KO23, and KO34 HCT116 cell lines. **D)** Relative levels of *KLF4* mRNA by RT-qPCR in the xenografted tumors of WT, KO23, and KO34 HCT116 cell lines. **E)** *In silico* motif for TCFL5 (blue triangle) in *SOX2* promoter. CORE (yellow box) and SRR1 (white box) are represented. **F)** *In silico* motif for TCFL5 (blue triangle) in *KLF4* promoter. Results are expressed as mean ± SEM (t-test; *p < 0.05, **p < 0.01, ***p<0.001; three independent experiments).

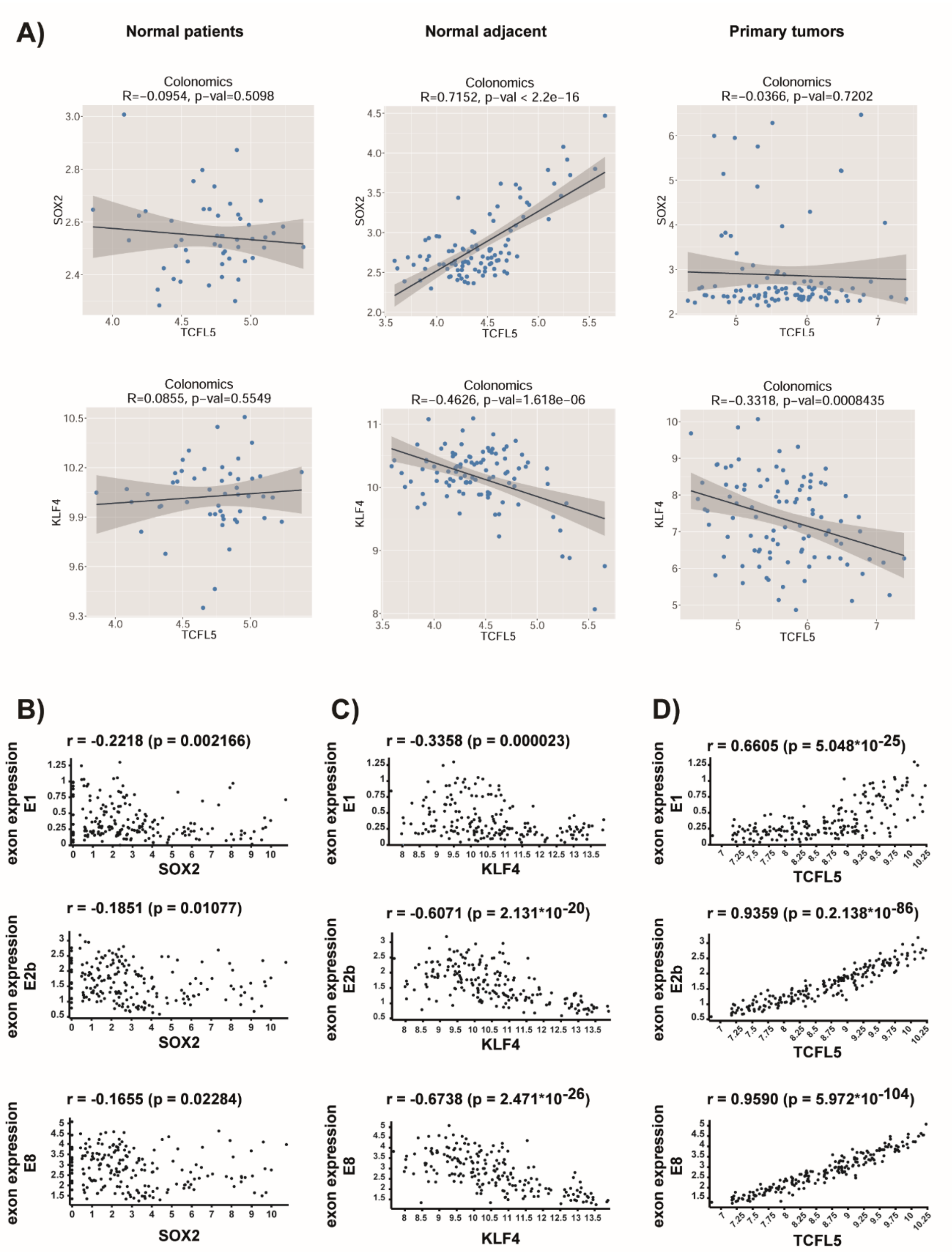

Figure S5. ***TCFL5*** **gene expression correlates with** ***SOX2*** **and** ***KLF4*** **in human colorectal cancer.** **A)** Correlation analysis of *SOX2*/*TCFL5* and *KLF4*/*TCFL5* in human CRC samples obtained from Colomics GSE44076 dataset, using Cancertool. Samples include non-cancer patient colon tissue, normal colon tissue adjacent to colon tumors, and colon tumor tissue. **B)** Pearson´s correlation analysis between exon E1, E2b, or E8 expression against *SOX2* gene expression using the data

from the TCGA Colon Cancer dataset on Xena browser. **C)** Pearson´s correlation analysis between exon E1, E2b, or E8 expression against *KLF4* gene expression using the data from the TCGA Colon Cancer dataset on Xena browser. **D)** Pearson´s correlation analysis between exon E1, E2b, or E8 expression against *TCFL5* gene expression using the data from the TCGA Colon Cancer dataset on Xena browser.

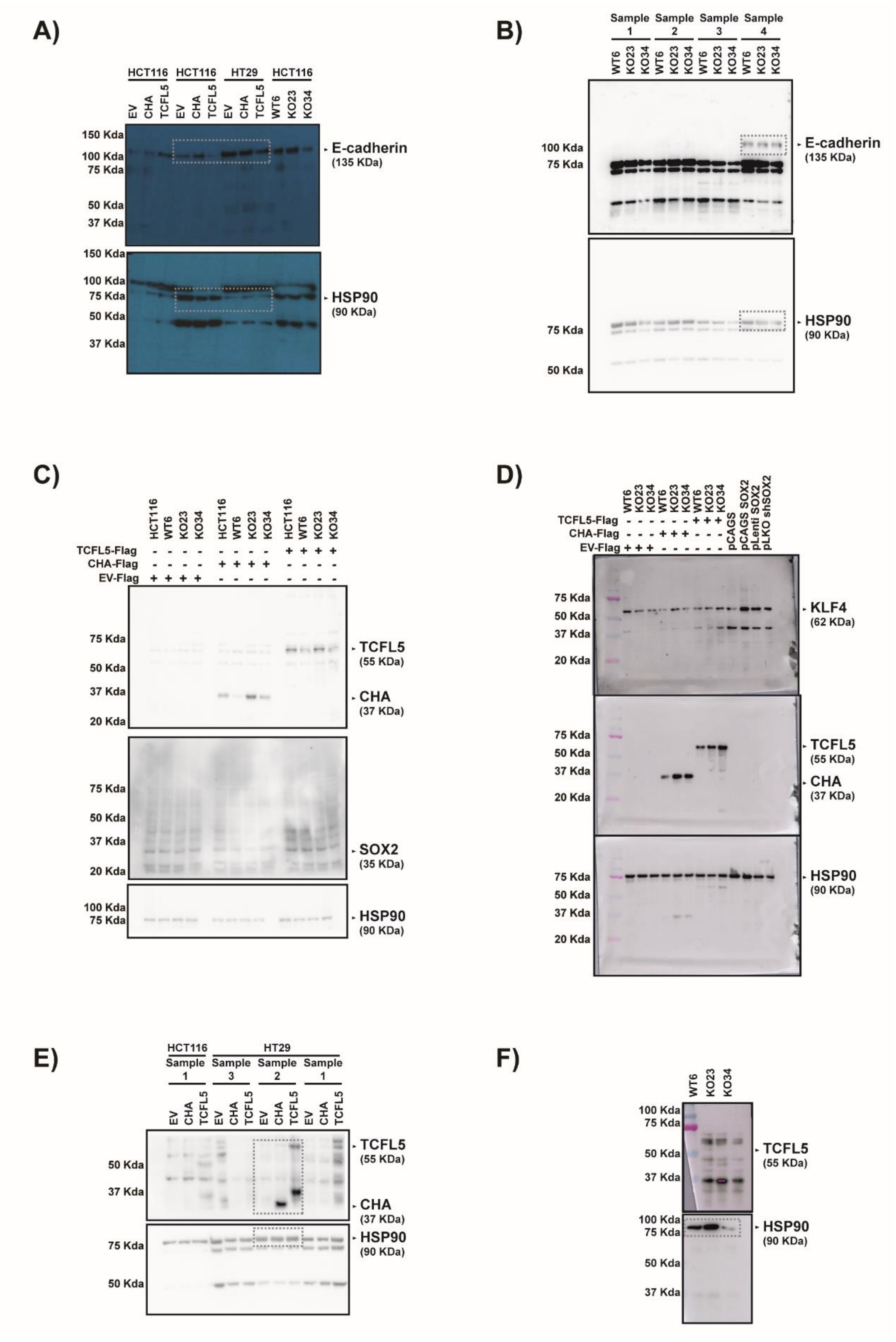

Figure S6. Complete membranes of Western-blot. Complete membranes of WB corresponding to **A)** Figure 3B, **B)** Figure 3D, **C)** Figure 5E, **D)** Figure 5F, **E)** Figure S3E, and **F)** Figure S3G.

TCFL5 oligonucleotides - exon junctions

Exon Junction	Sense (5' – 3')	Antisense (5' – 3')
E1/E2	ACCGCTTCAACAGCATCC	GATGGATGTCGAATGAGAGTTACC
E2/E3	CCCACTGTTTACTACAAATGCT	CTCTAGGCAATCCAATATCCTG
E2b/E3	GACTTCCACAGCAGCGATAG	CTCTAGGCAATCCAATATCCTG
E3/E4	GAGCAAGTTTGGATTAAAGTGGG	ACTGACGCATTCTACTCCGA
E4/E5	GAGAGGCATAACCGAATGGA	GCAGAACGGCACTAAGAG
E5/E6	CACAGCATTCCTGAAATACATCC	GCAGAAGTATATTCACCGAGTCC
E6/E7	GTATTCTGATCGGATCGTTTATGG	CATGACGTATCTGGAGTTTGTG
E7/E8	GAATCAGTTGAACCCAGGAG	GTCTGGTCAGCTTTAGCCT
E5/E7	CACAGCATTCCTGAAATACATCC	GGATTCTTTCCATCAACTGCT
E5/E8	CACAGCATTCCTGAAATACATCC	GTCTGGTCAGCTTTAGCCT

Oligonucleotides – qPCR

Gen	Sense (5' – 3')	Antisense (5' – 3')
GAPDH	GCACAAGAGGAAGAGAGAGACC	AGGGGAGATTCAGTGTGGTG
SOX2	GGTTCAGAAGCGAAAGCAGT	CCGCATCTCTTCCTCACG
KLF4	*GTGGAGAAAGATGGGAGC*	*TGACTTTGGGGTTCAGGT*
TCFL5/CHA	CACAGCATTCCTGAAATACATCC	GTCTGGTCAGCTTTAGCCT
TCFL5_E7	CACAGCATTCCTGAAATACATCC	GGATTCTTTCCATCAACTGCT
TCFL5_E6	CACAGCATTCCTGAAATACATCC	GCAGAAGTATATTCACCGAGTCC

Oligonucleotides – CHIP

Promoter	Sense (5' – 3')	Antisense (5' – 3')
CORE_01	AGCTGAGTTGGACAGGGAGA	CAGGTGCCAACACTCTCTCA
CORE_02	GCGTCCCATCCTCATTTAAG	AGCAACAGGTCACACCACAC
SRR1_01	CACACTCCTCTTCCCCTTGA	GAGAATGCTTCCAGCCAGAG
SRR1_02	GGTGGTCGTCAAACTCTGCT	TTGAGTGTGTTCCCCTCCTC
KLF4_positive	ATTATCCGCGTGACTCATCC	CCTCCTCTCCACACCCCTA
KLF4_negative	TCTCCCTCCAGGAAAAGGAG	CCTGAAGCCTGCATTCTAGC

Oligonucleotides – Cloning

Gen	Sense (5' – 3')	Antisense (5' – 3')
CHA	AAGGATCCATGAATGTTCCTCTTCAGCAAC<br>AAAACAAATG	GTTCTAGATCACTTGATCTCCATCGAG
TCFL5	CAGGATCCATGTCGGGCCCCGGAC	GTTCTAGATCACTTGATCTCCATCGAG

CRISPR guides

Name	Sequence (5' – 3') + ProtoSpacer
E3 A	TGGAAAGGACGAAACACCACCTGGTTAATTTTCGTACGGTTTAAGAGCTATGCTGGAAAC
E3 B	TGGAAAGGACGAAACACCACCCCGTACGAAAATTAACCGTTTAAGAGCTATGCTGGAAAC
E3 C	TGGAAAGGACGAAACACCCACCTGGTTAATTTTCGTACGTTTAAGAGCTATGCTGGAAAC
E4 A	TGGAAAGGACGAAACACCCACCTGGTTAATTTTCGTACGTTTAAGAGCTATGCTGGAAAC
E4 B	TGGAAAGGACGAAACACCGTGTGAGAACGTTGCGTTTGGTTTAAGAGCTATGCTGGAAAC
E4 C	TGGAAAGGACGAAACACCTATGTTATACTCGATGAGTGGTTTAAGAGCTATGCTGGAAAC

Silencing sequences

Name	Sequence (5' – 3')
shRNA TCFL5	GAATCCACTAAACAGACGTTACTCGAGTAACGTCTGTTTAGTGGATTC
siRNA TCFL5	CGACAUCCAUCUGAACUAA
siRNA control	CGACAUGAAAUUGCACUAA

Supplementary Table 1. Sequences of oligonucleotides. Sequences of restriction enzymes or CRISPR guides are underlined.

**Colorectal Adenocarcinoma (TCGA, PanCancer Atlas)**		
**Alteration**	**Gene alterated / total samples**	**Percentage (%)**
Missense mutation	6 / 524	1%
Amplification	12 / 524	2%
mRNA High	168 / 524	32%
Amplification + mRNA High	23 / 524	4%
Amplification + Missense mutation	3 / 524	1%
Total	212 / 524	40%

Supplementary Table 2. TCFL5 gene alteration. TCFL5 alterations were found in the PanCancer Atlas dataset. Alterations studied were missense mutation, amplification, and mRNA high. The number of cases of the total and percentage is represented in the table.

Exon E8 expression

	TP53		APC		PI3K		PTEN		KRAS	
	Samples (%)		Samples (%)		Samples (%)		Samples (%)		Samples (%)	
	WT	Mut	WT	Mut	WT	Mut	WT	Mut	WT	Mut
**High**	10 (92%)	109 (92%)	10 (8%)	109 (92%)	101 (85%)	18 (15%)	118 (99%)	1 (1%)	79 (66%)	40 (34%)
**Medium**	32 (27%)	87 (73%)	24 (20%)	95 (80%)	86 (72%)	33 (28%)	111 (93%)	8 (7%)	58 (49%)	61 (51%)
**Low**	72 (61%)	47 (39%)	45 (38%)	74 (62%)	76 (64%)	43 (36%)	102 (86%)	17 (14%)	58 (49%)	61 (51%)
**p-value**	<0.001		<0.001		<0.01		<0.01		<0.05	

Exon E1 expression

	TP53		APC		PI3K		PTEN		KRAS	
	Samples (%)		Samples (%)		Samples (%)		Samples (%)		Samples (%)	
	WT	Mut	WT	Mut	WT	Mut	WT	Mut	WT	Mut
**High**	16 (13%)	103 (87%)	12 (10%)	107 (90%)	94 (78%)	25 (21%)	117 (98%)	2 (2%)	71 (60%)	48 (40%)
**Medium**	40 (34%)	79 (66%)	32 (27%)	87 (73%)	86 (72%)	33 (28%)	107 (90%)	12 (10%)	67 (84%)	52 (44%)
**Low**	61 (51%)	58 (49%)	38 (32%)	81 (68%)	84 (71%)	35 (29%)	107 (90%)	12 (10%)	99 (83%)	(63 (53%)
**p-value**	<0.001		<0.001		ns		<0.05		ns	

Exon E2b expression

	TP53		APC		PI3K		PTEN		KRAS	
	Samples (%)		Samples (%)		Samples (%)		Samples (%)		Samples (%)	
	WT	Mut	WT	Mut	WT	Mut	WT	Mut	WT	Mut
**High**	11 (9%)	108 (91%)	10 (8%)	109 (92 %)	101 (85%)	18 (15%)	118 (99%)	1 (1%)	77 (65%)	42 (35%)
**Medium**	32 (27%)	87 (73%)	31 (26%)	88 (74%)	83 (70%)	36 (30%)	109 (92%)	10 (8%)	63 (53%)	56 (47%)
**Low**	75 (63%)	44 (37%)	41 (34%)	78 (66%)	79 (66%)	40 (34%)	104 (87%)	15 (13%)	54 (45%)	65 (55%)
**p-value**	<0.001		<0.001		<0.01		<0.01		<0.05	

Supplementary Table 3. *TCFL5* expression correlates with *TP53* and *APC* mutations. Gene/exon expression, mutation, and clinicopathological data from the TCGA Colon Cancer (COAD) collection were extracted using the UCSC Xena Browser analysis web tool. Specific TCFL5 gene or exon expression was calculated for exons E2b (chr20:61491477-61492109:-) and E1 (chr20:61492376-61493115:-). Patient samples were distributed in three groups according to TCFL5 gene or exon E1 or E2b expression, as high, medium, or low, and the percentages of patients with wild type (WT) or mutated (Mut) *TP53*, *APC*, *PIK3CA*, or *PTEN* genes were calculated. The statistical method used was the Chi-Square test comparing WT with the mutant form of the genes.
